# Embellicines C-E: Macrocyclic Alkaloids with a Cyclopenta[b]fluorene Ring System from the Fungus *Sarocladium* sp.

**DOI:** 10.1021/acs.jnatprod.2c01048

**Published:** 2023-03-08

**Authors:** Zeinab Y. Al Subeh, Laura Flores-Bocanegra, Huzefa A. Raja, Joanna E. Burdette, Cedric J. Pearce, Nicholas H. Oberlies

**Affiliations:** † Department of Medicinal Chemistry and Pharmacognosy, Faculty of Pharmacy, Jordan University of Science and Technology, Irbid 22110, Jordan; ‡ Department of Chemistry and Biochemistry, University of North Carolina at Greensboro, Greensboro 27402, North Carolina, United States; § Department of Pharmaceutical Sciences, University of Illinois at Chicago, Chicago 60612, Illinois, United States; ∥ Mycosynthetix, Inc., Hillsborough 27278, North Carolina, United States

## Abstract

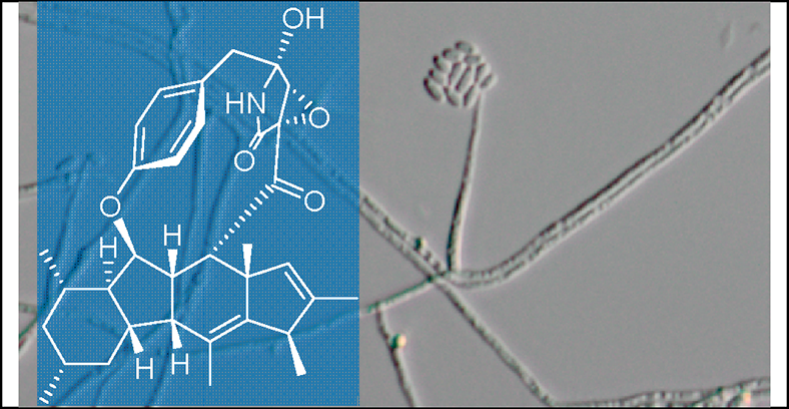

Macrocyclic alkaloids with a cyclopenta[b]fluorene ring system are a relatively young structural class of fungal metabolites, with the first members reported in 2013. Bioassay-guided fractionation of a *Sarocladium* sp. (fungal strain MSX6737) led to a series of both known and new members of this structural class (**1**–**5**), including the known embellicine A (**1**), three new embellicine analogues (**2**, **4**, and **5**), and a semisynthetic acetylated analogue (**3**). The structures were identified by examining both high-resolution electrospray ionization mass spectrometry data and one-dimensional and two-dimensional NMR spectra. The relative configurations of these molecules were established via ^1^H–^1^H coupling constants and nuclear Overhauser effect spectroscopy, while comparisons of the experimental electronic circular dichroism (ECD) spectra with the time-dependent density functional theory ECD calculations were utilized to assign their absolute configurations, which were in good agreement with the literature. These alkaloids (**1**–**5**) showed cytotoxic activity against a human breast cancer cell line (MDA-MB-231) that ranged from 0.4 to 4.8 μM. Compounds **1** and **5** were also cytotoxic against human ovarian (OVCAR3) and melanoma (MDA-MB-435) cancer cell lines.

Cancer is still the second leading cause of death worldwide despite recent improvements in its diagnosis and treatment.^1,2^ The prevalence of drug-resistant malignancies requires continual efforts to discover and develop new anticancer leads.^3−5^ Filamentous fungi are a promising source for potential anticancer leads,^6−8^ as they have yielded compounds with novel structures, new mechanisms of actions, and/or selective biological activities.^6,9,10^

Alkaloids, in particular, are a highly diverse class of natural products that exhibit a wide range of pharmacological properties.^11−13^ The *Dictionary of Natural Products* reports the isolation of more than 30 000 alkaloids from various natural sources.^14^ However, alkaloids with a cyclopenta[b]fluorene (6/5/6/5) ring system are relatively new to the literature. To date, only ten have been reported, and all of those have been isolated from endophytic fungi.^14^ Embellicines A and B, reported in 2013, have a tetracyclic core fused with a 13-membered macrocycle,^15^ and their cytotoxic properties were purported to be due to TNFα-induced NF-κB transcriptional activity. Phomapyrrolidones A–C were reported concurrently and found to exhibit weak antitubercular activity.^16^ Recently, ascomylactams A–C^17^ and didymellanosine^18^ were characterized and reported to exhibit cytotoxic and antimicrobial activities. Didymellanosine is the first 13-membered macrocyclic alkaloid with a cyclopenta[b]fluorene ring system conjugated with adenosine.^18^ A structurally related group of compounds, hirsutellones,^19−21^ pyrrocidines,^22−24^ and pyrrospirones,^25,26^ have a decahydrofluorene skeleton and gained considerable attention^21,27^ for their unique core and pharmacological activities as antimicrobial and cytotoxic leads.

In the course of ongoing studies to discover fungal metabolites with potent cytotoxic activities,^28−32^ we report the characterization of a series of embellicines (**1**–**5**), including the isolation of the known embellicine A (**1**), three new embellicines C, D, and E (**2**, **4**, and **5**, respectively), and the semisynthetic generation of the acetylated analogue, 2′-*O*-acetyl-embellicine C (**3**). These macrocyclic alkaloids with a cyclopenta[b]fluorene ring system were obtained from the organic extract of a *Sarocladium* sp. (fungal strain MSX6737), which exhibited cytotoxic activity against human melanoma, breast, and ovarian cancer cells when tested at a concentration of 20 μg/mL (i.e., less than 40% cancer cell survival relative to a negative control).^32^ The structures were determined by examining their one-dimensional (1D) and two-dimensional (2D) NMR data. In addition, nuclear Overhauser effect spectroscopy (NOESY) spectra, along with the experimental and calculated ECD spectra, were used to assign their relative and absolute configurations.

## Results and Discussion

Fungal strain MSX6737 was cultured on a solid rice medium for 3 weeks, and extraction and bioactivity-directed fractionation afforded compounds **1**, **2**, **4**, and **5**. The taxonomy of this strain was examined using both morphological and molecular methods, indicating that it is likely a new *Sarocladium* sp., and although it was obtained as a saprobe from leaf litter, many other members of this genus are reported as plant pathogens, opportunistic human pathogens, endophytes, and mycoparasites.^33^ As it may represent a new fungal species,^34^ further taxonomic studies on strain MSX6737 are ongoing.
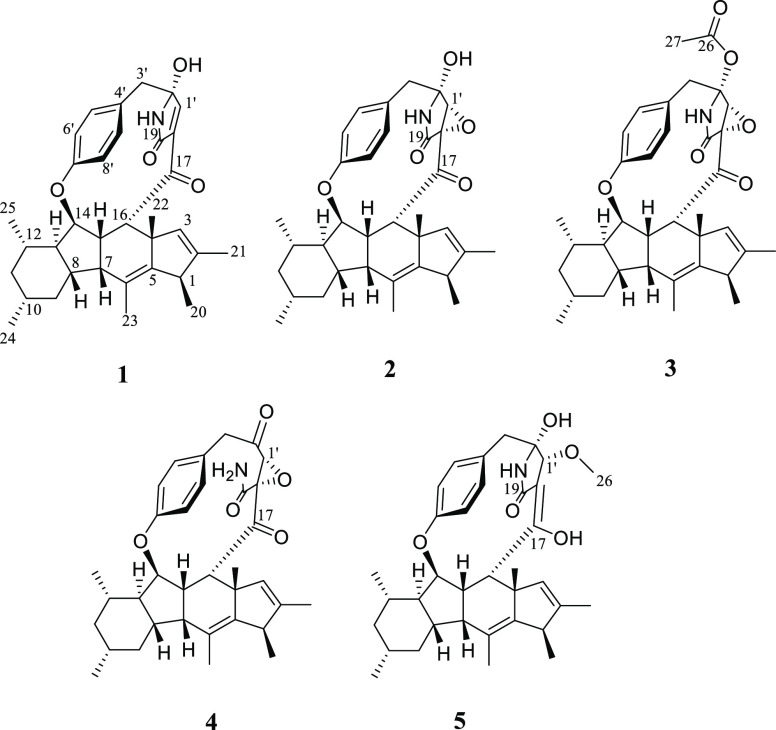


The molecular formula of compound **1** was deduced as C_34_H_41_NO_4_ via high-resolution electrospray ionization mass spectrometry (HRESIMS), indicating an index of hydrogen deficiency of 15. ^1^H and ^13^C NMR data (Figure S2 and Table S1) indicated the presence of six methyl, two carbonyl, three methylene, six aromatic, and six olefinic carbons. Searching the *Dictionary of Natural Products* for compounds with a similar molecular formula, compound **1** was identified as embellicine A based on the matched NMR data (Table S1) and ECD spectrum (Figure S27). Embellicine A was reported in 2013 by Ebrahim et al. as an alkaloid with a cyclopenta[b]fluorene (6/5/6/5) ring system fused with a 13-membered macrocycle mediated by the presence of a γ-lactam moiety and a *para*-substituted benzene with restricted free rotation.^15^ Embellicine A exhibits a double bond between C-1′ and C-18, as suggested by the ^13^C signals at δ_C_ 157.8 and 131.1 and the olefinic proton at δ_H_ 7.14. The absolute configuration of embellicine A (**1**) was previously reported as 1*R*, 4*S*, 7*R*, 8*S*, 10*R*, 12*S*, 13*R*, 14*R*, 15*S*, 16*S*, 2′*R* as established via time-dependent density functional theory (TDDFT) ECD calculations.^15^ Later, ascomylactam B was isolated from *Didymella* sp. and found to share the same planar structure of embellicine A (**1**); however, ascomylactam B differs from **1** in the configurations at C-1, C-4, C-7, C-14, and C-16.^17^ Comparison of the ECD spectra and optical rotations between embellicine A and ascomylactam B were reported previously.^15,17^

**Figure 1:**
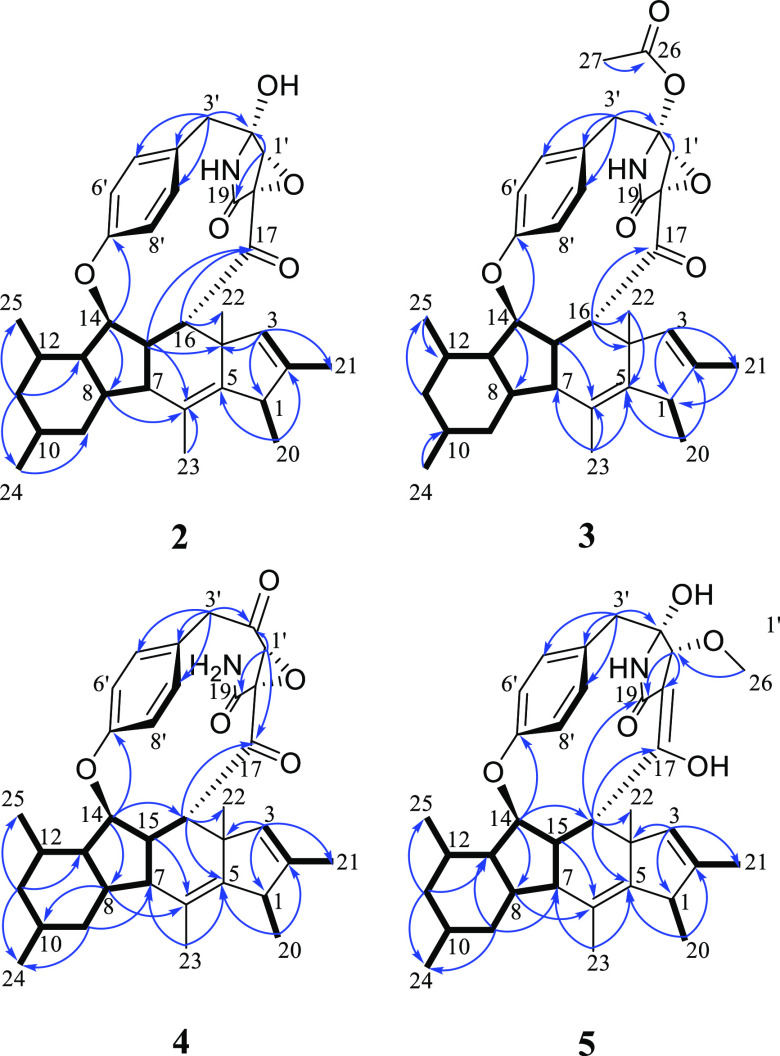
Key COSY (bold lines) and HMBC (arrows) correlations of compounds **2**–**5**.

The molecular formula of compound **2**, which was obtained as a white amorphous powder, was C_34_H_41_NO_5_, as deduced via HRESIMS, indicating an index of hydrogen deficiency of 15 (Figure S1). The ^1^H and ^13^C NMR data (Tables 1 and 2) presupposed structural similarities with embellicine A (**1**). Correlation spectroscopy (COSY) and heteronuclear multiple-bond coherence (HMBC) correlations of the four doublet of doublet protons resonating at δ_H_ 6.96, 6.99, 7.16, and 7.24 suggested a *para*-substituted aromatic ring with restricted rotation (Figures S4–S6). The presence of a tetracyclic cyclopenta[b]fluorene (6/5/6/5) ring system was deduced by three proton spin systems (Figure 1). The first spin system extended from H-7 to H-16, the second was observed between H_3_-20 and H-1, and the third consisted of the allylic coupling between H_3_-21 and the olefinic proton at C-3. The six methyl groups attached to the tetracyclic ring system were positioned based on their COSY and HMBC correlations (Figure 1). Carbons resonating at δ_C_ 63.3 for C-1′, 60.0 for C-18, 84.9 for C-2′, and 166.5 for the amide carbonyl (C-19) suggested the presence of a γ-lactam moiety, similar to that reported in ascomylactam C and phomapyrrolidone C.^16,17^ This was confirmed by the HMBC correlations of H-1′ with C-2′ and C-19 (Figure 1). The chemical shifts of the two adjacent carbons, C-1′ and C-18, were more shielded as compared to the hydroxylated carbon at C-2′, indicating they were part of an oxirane.^16,17^ The HMBC correlations of H_2_-3′ with the aromatic carbons (C-4′, C-5′, and C-9′) and the hemiaminal carbon (C-2′) suggested that C-3′ intermediates the connection between the *para*-substituted benzene and the γ-lactam (Figure 1). On the other hand, the HMBC correlation of H-14 with the oxygenated aromatic carbon (C-7′) confirmed the ether linkage between the *para*-substituted benzene and the tetracyclic ring system. The linkage between the γ-lactam moiety and the tetracyclic ring system through the C-17 keto group was established based on the HMBC correlations of H-15 and H-16 with C-17 (Figure 1). Both phomapyrrolidone C and ascomylactam C were found to share the same planar structure as **2**.^16,17^ However, for both of these compounds neither the NMR data (Tables S2 and S3) nor the ECD spectra (Figure S9) matched those of **2**, suggesting a diastereomeric relationship between these three compounds. The absolute configuration of phomapyrrolidone C, deduced via analysis of NOESY spectra and ECD calculations, was recently reported as 1*S*, 4*R*, 7*S*, 8*S*, 10*R*, 12*S*, 13*R*, 14*R*, 15*S*, 16*S*, 18*R*, 1′*R*, 2′*R* and was shown to be the 16*S*-epimer of ascomylactam C.^17^

**Table 1:** ^1^H NMR Spectroscopic Data for 2–4 in CDCl_3_ and 5 in Acetone-*d*_6_

	**2**^a^	**3**^b^	**4**^a^	**5**^b^
position	δ_H_ (*J*, Hz)	δ_H_ (*J*, Hz)	δ_H_ (*J*, Hz)	δ_H_ (*J*, Hz)
1	2.98, q (7.0)	2.98, q (7.1)	3.03, q (7.0)	3.05, q (6.9)
3	5.15, br s	5.15, m	5.29, br s	5.16, m
7	2.84, m	2.84, m	2.88, m	2.96, m
8	1.95, m	1.94, m	1.96, m	1.99, m
9a	1.90 m	1.90, m	1.92, m	1.97, m
9b	1.48, ddd (11.9, 11.9, 11.9)	1.49, ddd (11.9, 11.9, 11.9)	1.48, m	1.56, ddd (11.3, 11.3, 11.3)
10	1.40 m	1.41, m	1.42, m	1.40, m
11a	1.69 m	1.70, m	1.72, m	1.70, m
11b	0.72, ddd (12.0, 12.0, 12.0)	0.72, ddd (12.0, 12.0, 12.0)	0.73, ddd (12.0, 12.0, 12.0)	0.76, ddd (12.1, 12.1, 12.1)
12	1.82, m	1.80, m	1.86, m	1.89, m
13	1.22, ddd (13.6, 10.4, 7.7)	1.23, ddd (13.7, 10.4, 7.8)	1.28, ddd (13.6, 10.2, 7.8)	1.19, ddd (13.5, 10.4, 7.4)
14	4.72, dd (7.7, 3.7)	4.80, dd (7.8, 3.8)	4.95, dd (7.8, 4.0)	4.74, dd (7.4, 3.8)
15	2.45, ddd (10.2, 6.9, 3.7)	2.43, ddd (10.2, 6.7, 3.8)	2.43, ddd (10.2, 7.3, 4.0)	2.81, m
16	2.39, d (10.2)	2.39, d (10.2)	2.57, d (10.2)	2.55, d (10.9)
20	0.97, d (7.0)	0.97, d (7.1)	0.94, d (7.0)	1.04, d (6.9)
21	1.55, br s	1.53, br s	1.55, br s	1.51, br s
22	1.00, s	1.01, s	1.06, s	1.05, s
23	1.81, br s	1.81, br s	1.83, br s	1.84, br s
24	0.95, d (6.5)	0.95, d (5.74)	0.96, d (6.1)	0.97, d (6.5)
25	1.07, d (6.4)	1.07, d (6.4)	1.09, d (6.4)	1.12, d (6.4)
26				3.44, s
27		2.17, s		
1′	3.38, d (2.4)	3.69, d (2.6)	2.95, s	3.64, s
3′a	3.31, d (13.6)	3.61, d (13.4)	3.83, d (18.2)	3.23, d (12.9)
3′b	3.15, d (13.6)	3.23, d (13.4)	3.65, d (18.2)	2.95, d (12.9)
5′	7.16, dd (8.4, 2.0)	7.14, dd (8.5, 2.2)	7.51, dd (8.4, 2.1)	7.11, dd (8.4, 2.1)
6′	6.96, dd (8.4, 2.3)	6.96, dd (8.5, 2.5)	7.04, dd (8.4, 2.4)	6.82, dd (8.4, 2.4)
8′	6.99, dd (8.1, 2.3)	7.00, dd (8.1, 2.5)	7.00, dd (8.1, 2.4)	7.07, dd (8.1, 2.4)
9′	7.24, dd (8.1, 2.0)	7.28, dd (8.1, 2.2)	7.29, dd (8.1, 2.1)	7.30, dd (8.1, 2.1)
19-NH	5.87, br s	6.31, d (2.3)	5.40, br s	7.24, br s
			5.68, br s	
2′–OH				4.64, br s
17-OH				12.18, s
5-OH				

a Recorded at 400 MHz.

b Recorded at 500 MHz.

**Table 2:** ^13^C NMR Spectroscopic Data for 2–4 in CDCl_3_ and 5 in Acetone-*d*_6_

	**2**^b^	**3**^a^	**4**^b^	**5**^a^
position	δ_C_, type	δ_C_, type	δ_C_, type	δ_C_, type
1	44.7, CH	44.7, CH	44.9, CH	45.5, CH
2	143.1, C	143.2, C	144.3, C	140.0, C
3	129.1, CH	128.9, CH	129.3, CH	132.4, CH
4	52.9, C	53.0, C	54.0, C	51.6, C
5	147.6, C	147.8, C	146.5, C	149.2, C
6	129.3, C	129.2, C	130.2, C	128.1, C
7	44.6, CH	44.6, CH	44.6, CH	45.3, CH
8	46.3, CH	46.3, CH	46.4, CH	47.1, CH
9	38.4, CH_2_	38.3, CH_2_	38.4, CH_2_	39.4, CH_2_
10	33.8, CH	33.8, CH	33.9, CH	34.7, CH
11	44.3, CH_2_	44.4, CH_2_	44.4, CH_2_	45.0, CH_2_
12	31.9, CH	31.9, CH	32.0, CH	32.4, CH
13	50.7, CH	50.7, CH	50.9, CH	51.8, CH
14	88.1, CH	87.9, CH	87.3, CH	91.5, CH
15	51.5, CH	51.5, CH	51.3, CH	52.9, CH
16	51.3, CH	51.2, CH	48.4, CH	48.9, CH
17	199.0, C	198.9, C	200.9, C	170.6, C
18	60.0, C	58.3, C	67.0, C	105.3, C
19	166.4, C	165.4, C	164.1, C	174.8, C
20	17.4, CH_3_	17.4, CH_3_	16.9, CH_3_	18.5, CH_3_
21	15.1, CH_3_	15.0, CH_3_	15.0, CH_3_	15.1, CH_3_
22	26.4, CH_3_	26.4, CH_3_	25.7, CH_3_	26.8, CH_3_
23	20.0, CH_3_	19.9, CH_3_	19.8, CH_3_	20.4, CH_3_
24	22.8, CH_3_	22.8, CH_3_	22.8, CH_3_	23.0, CH_3_
25	20.7, CH_3_	20.7, CH_3_	20.7, CH_3_	20.9, CH_3_
26		170.5, C		59.5, CH_3_
27		21.5, CH_3_		
1′	63.3, CH	62.0, CH	61.5, CH	81.5, CH
2′	84.9, C	89.1, C	201.3, C	89.4, C
3′	44.3, CH_2_	43.9, CH_2_	48.4, CH_2_	46.9, CH_2_
4′	128.6, C	127.8, C	127.3, C	131.0, C
5′	130.5, CH	130.5, CH	135.4, CH	131.7, CH
6′	123.8, CH	124.1, CH	123.2, CH	123.2, CH
7′	159.3, C	159.5, C	159.9, C	161.0, C
8′	122.6, CH	122.7, CH	122.2, CH	123.7, CH
9′	132.3, CH	132.4, CH	131.6, CH	133.4, CH

a Recorded at 100 MHz.

b Recorded at 125 MHz.

**Figure 2:**
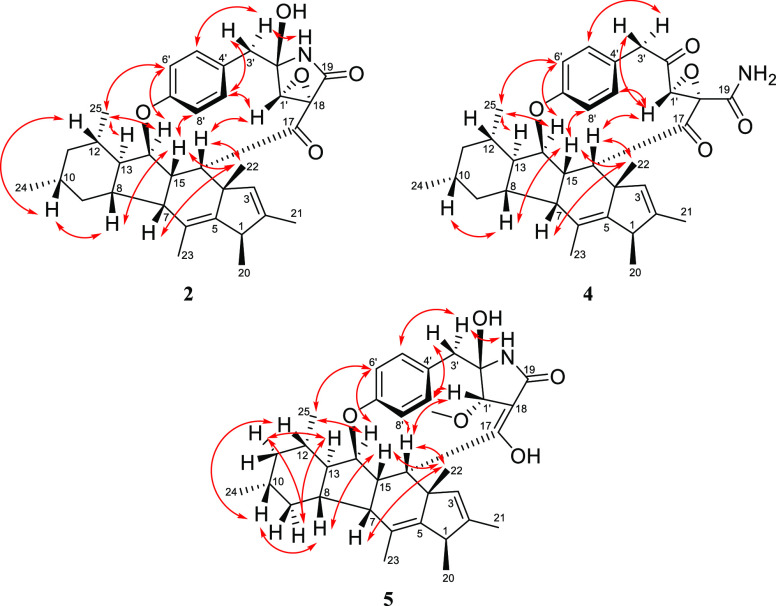
Key NOESY correlations of **2**, **4**, and **5**.

The NOESY spectrum, along with the ^1^H–^1^H coupling constants, were analyzed to assign the relative configuration of the asymmetric centers in **2** (Figure 2). The large diaxial coupling constant of H-9b with both H-8 and H-10 (^*3*^*J*_H-9b, H-8_ = ^*3*^*J*_H-9b, H-10_ = 11.9 Hz) and the coupling of H-11b with H-10 and H-12 (^*3*^*J*_H-11b,H-10_ = ^*3*^*J*_H-11b,H-12_ = 12.0 Hz) indicated that H-8, H-10, and H-12 were cofacial and in axial orientations, while CH_3_-24 and CH_3_-25 were in equatorial orientations (Table 1) and syn to each other (Figure S7). This was further confirmed by the NOESY cross-peak of H-10 with H-12 (Figure 2). The relative configuration of the tetracyclic ring system was assigned based on the observed NOESY correlations of H-7/H_3_-22, H_3_-22/H-16, H_3_-22/H-15, H-15/H-8, H-8/H-10, and H-10/H-12, which supported the cofacial orientation of H-7, H-8, H-10, H-12, H-15, H-16, and CH_3_-22 (Figures 2 and S8). The NOESY correlations between H-13/H_3_-25/H-14 indicated their cofacial relationship, which was presumed to be opposite to those stated above. The configuration at C-1 was challenging to assign due to the close chemical shift values of the two methyls CH_3_-20 and CH_3_-22. Accordingly, NOESY correlation between H_3_-20 and H_3_-22 could not be recognized within the high noise level around the diagonal axis in the spectrum (Figure S7). However, these could be cofacial based on biogenetic considerations, as all previously described fungal metabolites with a cyclopenta[b]fluorene (6/5/6/5) ring system have CH_3_-20 and CH_3_-22 oriented on the same face, including embellicine A (**1**).^16−18^ The relative configuration of H-1′ was assigned based on its cross-peak with H-16 and H-9′. The restricted rotation of the benzene ring inside the 13-membered macrocycle was further supported by the NOESY correlation of H-15 with H-8′, while H_3_-25 and H-14 correlated with H-6′. The orientation of the γ-lactam was established based on the NOESY correlation of the NH proton with H-3a′ (Figure 2). Therefore, the relative configuration of **2** was assigned as 1R*, 4S*, 7R*, 8S*, 10R*, 12S*, 13R*, 14R*, 15S*, 16S*, 18R*, 1′R*, 2′R*.

**Figure 3:**
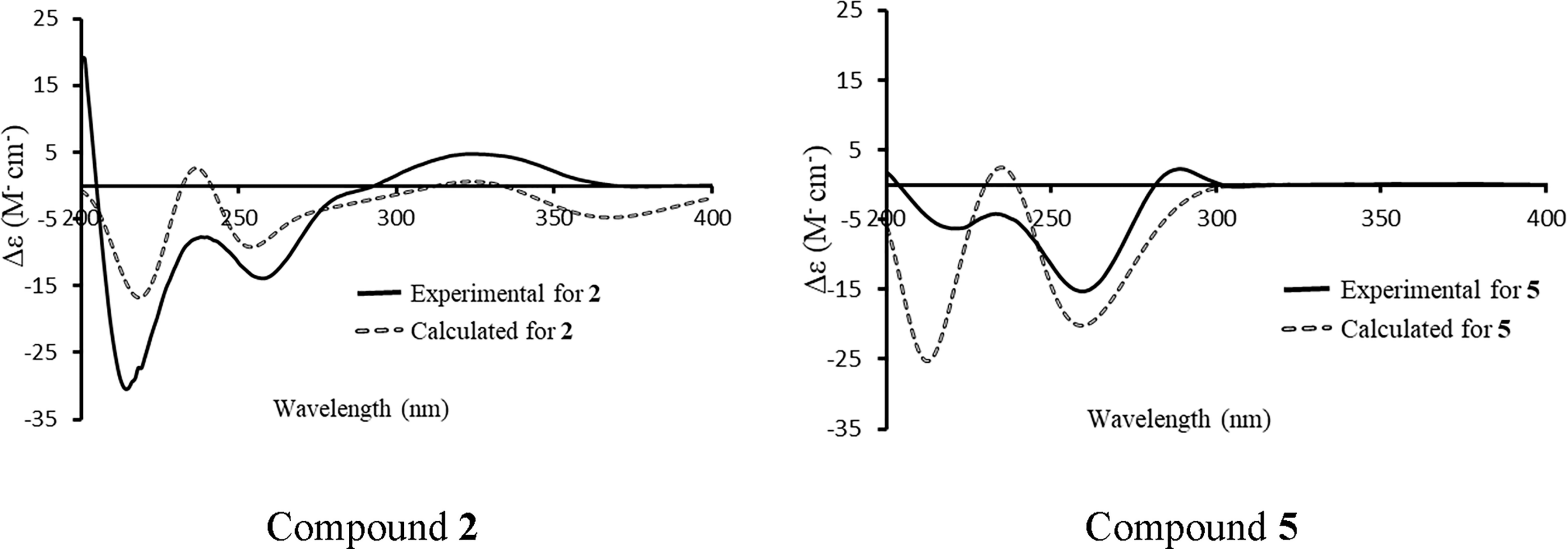
Experimental vs calculated ECD spectra of **2** and **5** in CH_3_CN.

Theoretical calculations of electronic circular dichroism (ECD) spectra have emerged as a promising tool for determining the absolute configuration of chiral natural products,^35^ and previous studies by our team have used these to establish the absolute configuration of several fungal metabolites.^29,36,37^ The time-dependent DFT (TDDFT) method with the B3LYP/6-31G+(d) level of theory was used to calculate the theoretical ECD spectra for the suggested configuration of **2** (1*R*, 4*S*, 7*R*, 8*S*, 10*R*, 12*S*, 13*R*, 14*R*, 15*S*, 16*S*, 18*R*, 1′*R*, 2′*R*), which matched well with its experimental ECD spectrum (Figure 3). Accordingly, the absolute configuration of **2** was identified as 1*R*, 4*S*, 7*R*, 8*S*, 10*R*, 12*S*, 13*R*, 14*R*, 15*S*, 16*S*, 18*R*, 1′*R*, 2′*R* and given the trivial name embellicine C. Compared to phomapyrrolidone C, the configurations at C-1, C-4, and C-7 were different in **2** (Figure S9);^17^ ascomylactam C has an added difference with **2** based on opposite configurations at positions C-1, C-4, C-7, and C-16 (Figure S9).

Acylation of **2** afforded the acetyl analogue at the 2′–OH position (**3**) with a molecular formula of C_36_H_43_NO_6_ as deduced via HRESIMS; this analogue was generated to further explore the structure–activity relationship (SAR) of these compounds. The two extra carbons at δ_C_ 170.5 and 21.5 and the singlet methyl at δ_H_ 2.17 supported this assignment (Tables 1 and 2). The NOESY spectrum of **3** showed similar correlations to those observed for **2**, which further supported the interpretations of the NOESY correlations observed in **2**. Their ECD spectra were also nearly identical (Figure S20), and given the relationship to **2**, this compound was ascribed the trivial name 2′-*O*-acetyl-embellicine C (**3**).

Compound **4** was found to have the same molecular formula as **2**, as indicated by HRESIMS (C_34_H_41_NO_5_), and ^1^H and ^13^C NMR data suggested the same tetracyclic system and aromatic ring as **2** (Tables 1 and 2). However, **4** showed an extra signal at δ_C_ 201.3 and was missing a signal at δ_C_ 84.9 (i.e., C-2′ in **2**), which suggested opening of the γ-lactam and the formation of a ketone at C-2′. COSY correlations between the two exchangeable protons at δ_H_ 5.40 and 5.68 supported the presence of an NH_2_ group and the lack of a bond between the amide nitrogen and C-2′. ^1^H–^1^H coupling constants and the NOESY spectrum of **4** suggested the same relative configuration as **2** (Figure 2). Major similarities in their experimental ECD spectra were also observed (Figure S20), and partial conversion of **2** into **4** was observed upon storage as a dry compound at room temperature, and vice versa (Figure S21). Thus, **4** shared the same absolute configuration as **2**, which was 1*R*, 4*S*, 7*R*, 8*S*, 10*R*, 12*S*, 13*R*, 14*R*, 15*S*, 16*S*, 18*R*, 1′*R*, and it was ascribed the trivial name embellicine D. Interestingly, the opening of the γ-lactam was not reported previously for the two diastereoisomers of **2**, namely, phomapyrrolidone C and ascomylactam C.^16,17^

Compound **5** was isolated as a white amorphous powder with a molecular formula of C_35_H_45_NO_5_ as determined via HRESIMS. Comparing the 1D and 2D NMR data of **5** with those observed for embellicine A (**1**) indicated the presence of an extra methoxy group attached to C-1′ (δ_C_ 59.5), while lacking the double bond between C-1′ and C-18. The HMBC correlation between H_3_-26 and C-1′ verified the connectivity of the methoxy group at C-1′ (Figure 1). Moreover, an intramolecular hydrogen bond and the proton observed at δ_H_ 12.18 suggested an enol moiety at C-17. The carbon signal at δ_C_ 170.6 for C-17 in **5**, instead of δ_C_ 195.4 in **1**, further supported this conclusion. The enol form at C-17 was reported previously for embellicine B,^15^ which is structurally similar to **5**.^15^ However, the methoxy group attached to C-1′ in **5** is a hydroxy in embellicine B. The NOESY spectrum of **5** indicated the same relative configuration for the tetracyclic rings observed in **1**–**4** (Figure 2). The orientation of H-1′ was established by its NOESY correlation with H-16 (Figure 2). The enol functional group in both **5** and embellicine B limited their stability, as conversion to **1** was noted both in the literature^15^ and in this study. Moreover, **5** is likely an artifact that results from exposing **1** to MeOH, which was used through the isolation and purification process.^38^ Accordingly, the absolute configuration of **5** was suggested as 1*R*, 4*S*, 7*R*, 8*S*, 10*R*, 12*S*, 13*R*, 14*R*, 15*S*, 16*S*, 1′*R*, 2′*R*, and it was ascribed the trivial name embellicine E. The calculated and experimental ECD data for **5** were in good agreement, further supporting the absolute configuration of this compound (Figure 3). Ascomylactam A is a diastereoisomer of **5**, as they share the same planar structure; however, the absolute configurations at C-1, C-4, and C-7 were different between these compounds.^17^

The purity of compounds **1**–**5** was >95%, as assessed by UPLC (Figure S28), prior to evaluation of cytotoxic activities against MDA-MB-231 (human breast cancer), OVCAR3 (human ovarian cancer), and MDA-MB-435 (human melanoma cancer) cell lines. Their half-maximal inhibitory concentration (IC_50_) values against breast cancer cells ranged between 0.4 and 4.8 μM (Table 3). Compounds **1** and **5** were also cytotoxic against both ovarian and melanoma cell lines (IC_50_ 1.0–1.8 μM), while compounds **2**–**4** showed less toxicity against these two cell lines (IC_50_ > 10 μM) (Table 3). Compounds **1** and **5** were more potent against the three cancer cell lines, and both lack the oxirane ring exhibited by **2**–**4**. Cytotoxic activities against various cancer cell lines were reported previously for the structurally related analogues, ascomylactams A–C, phomapyrrolidone A, and phomapyrrolidone C.^17^

**Table 3:** Cytotoxic Activities of 1–5 against Three Cancer Cell Lines

IC_50_ (μM)			
Compound	MDA-MB-231	OVCAR3	MDA-MB-435
**1**	0.4	1.6	1.5
**2**	4.8	21	17
**3**	4.4	20	20
**4**	3.2	24	18
**5**	0.4	1.0	1.8
taxol	0.6 × 10^–3^	1.8 × 10^–3^	0.3 × 10^–3^

The biosyntheses of embellicines, and similar analogues with a cyclopenta[b]fluorene (6/5/6/5) ring system, have been reported via polyketide synthase–nonribosomal peptide synthetase (PKS–NRPS) complexes.^21,27^ Unlike hirsutellones and pyrrocidines, which are derived from the polycyclization of a Tyr-C_18_ linear precursor, alkaloids with an additional five-membered cycle fused with ring A are hypothesized to derive from a Tyr-C_20_ intermediate (Figure S29).^21,27,39^ The five methyl moieties along the linear C_18_ chain could then be introduced from *S*-adenosylmethionine. It is suggested that the 13-membered ring, along with ring C of the cyclopenta[b]fluorene (6/5/6/5) ring system, results from connecting the tyrosine phenol with the acyclic polyketide chain. A subsequent Diels–Alder cyclization results in the formation of rings A and B. The five-membered cycle (i.e., ring D) is probably formed via electrophilic cyclization of the diene intermediate.^21^ Interestingly, embellicines (**1**–**5**) share the same core in terms of structure and configurations, and these coisolated compounds differ only in the structure of the γ-lactam, where embellicine A (**1**) possesses a double bond between C-18 and C-1′ and, thus, may be the parent molecule of the embellicine class (Figure S29).

## Experimental Section

### General Experimental Procedures

Optical rotations were obtained using a Rudolph Research Autopol III polarimeter (Rudolph Research Analytical). UV and ECD spectra were collected using a Varian Cary 100 Bio UV–Vis spectrophotometer and an Olis DSM 17 ECD spectrophotometer, respectively. 1D and 2D NMR experiments were conducted using a JEOL ECA–500 NMR spectrometer operating at 500 MHz or a JEOL ECS-400 NMR spectrometer, operating at 400 MHz and equipped with a high-sensitivity JEOL Royal probe and a 24-slot autosampler. Residual solvent signals were used as an internal standard (For CDCl_3_ δ_H_/δ_C_ 7.26/77.16, acetone-*d*_6_ δ_H_/δ_C_ 2.05/29.8, and for deuterated dimethyl sulfoxide (DMSO-*d*_6_) δ_H_/δ_C_ 2.50/39.52). UPLC-HRESIMS data were collected via a Thermo Fisher Scientifc Q Exactive Plus mass spectrometer that is connected to Waters Acquity UPLC system. BEH Shield RP18 column (Waters, 1.7 μm; 50 × 2.1 mm) was used and heated to 40 °C. The mobile phase consisted of CH_3_CN-H_2_O (1% formic acid) in a gradient system of 15:85 to 100:0 over 10 min and at a flow rate of 0.3 mL/min. MS data were collected from 150 to 2000 *m*/*z* while alternating between positive and negative modes. The purity of compounds **1**–**5** was evaluated using a Waters Acquity UPLC system (Waters Corp.) utilizing a BEH Shield RP18 column (Waters, 1.7 μm; 50 × 2.1 mm). Data were collected and analyzed using Empower 3 software (Waters). All analytical and preparative high-performance liquid chromatography (HPLC) runs were carried out using a Varian Prostar HPLC system equipped with ProStar 210 pumps and a Prostar 335 photodiode array detector (PDA). HPLC data was collected and analyzed using Galaxie Chromatography Workstation software (version 1.9.3.2, Varian Inc.). For preparative HPLC, a Phenomenex Gemini-NX C_18_ (5 μm; 250 × 21.2 mm) was used, while a Phenomenex Luna-PFP (5 μm; 250 × 10 mm) was utilized for semipreparative HPLC. Flash chromatography was carried out using a Teledyne ISCO CombiFlash Rf 200 that was equipped with UV and evaporative light-scattering detectors.

### Fungal Strain Isolation and Identification

The fungal strain MSX6737 was isolated from tropical terrestrial leaf litter by Dr. Barry Katz (MYCOsearch, Inc.). To identify the fungal strain, both morphological and molecular analyses were employed. The micromorphological characters, such as narrowly cylindrical phialides arising from vegetative hyphae, tapering toward the apexes, producing abundant, one-celled, cylindrical, hyaline conidia in slimy heads (Figure S30), agree with the concept of the genus *Sarocladium.*(33,40−42) To corroborate morphological identification and to add an additional means of orthogonal data, the entire internal transcribed spacer region (ITS1, 5.8S, ITS2) as well as the partial large subunit (nrLSU-26S or 28S) were both polymerase chain reaction (PCR) amplified and sequenced using primer combinations ITS1F and ITS4^43,44^ for the former and LROR and LR6 for the latter;^45,46^ previously outlined protocols were utilized.^47^ The Sanger sequenced ITS region was BLAST searched using NCBI GenBank, showing ≥90% sequence homology with *Sarocladium,* while LSU BLAST search showed ≥98% sequence homology with members of *Sarocladium*. To better understand the phylogenetic affiliation of strain MSX6737 within the genus *Sarocladium,* a maximum likelihood phylogeny using combined ITS and LSU was inferred using IQ-TREE^48^ in the program PhyloSuite v.1.2.1.^49^ All previously described *Sarocladium* sequences were obtained from recent molecular phylogenetic studies.^33,40−42,50,51^ ModelFinder^52^ predicted GTR+I+G as the best fitting substitution model for both ITS and LSU regions according to the Akaike Information Criterion.^53^ The trimmed nucleotide alignment after removing ambiguous nucleotide positions with GBlocks^54,55^ was then used to run the maximum likelihood analysis using IQ-Tree using Ultrafast bootstrapping;^48,49,56^ only values ≥95% for the clades were considered strongly supported. The maximum likelihood analysis showed a distinct lineage within *Sarocladium,* suggesting that strain MSX6737 is a putative new species of *Sarocladium, Sarocladiacae, Hypocreales, Ascomycota* (Figure S31). Thus, based on morphology and molecular data, we refer to strain MSX6737 as *Sarocladium* sp. pending new species description in a mycology journal.^34^ The sequence data were deposited in GenBank with accession numbers: ITS: OP650545, OP650546; LSU: OP650548, OP650549.

### Extraction and Isolation

EtOAc (900 mL) was added to a large-scale solid fermentation culture of MSX6737 before being chopped with a spatula and shaken for ∼18 h at ∼100 rpm at rt. The sample was then filtered using vacuum, and the filtrate was evaporated to dryness. The dried extract was reconstituted in 300 mL of CH_3_CN and 200 mL of hexane. The biphasic solution was stirred for 30 min and then transferred to a separatory funnel. The CH_3_CN layer was drawn off and evaporated to dryness under vacuum. This defatted material (575 mg) was dissolved in CHCl_3_, adsorbed onto Celite 545, and then fractionated via flash chromatography using a gradient solvent system of hexane-EtOAc at a flow rate of 35 mL/min and 28 column volumes over 27 min to afford three fractions. The second fraction of flash chromatography was subjected to a preparative reverse-phase HPLC over a Phenomenex Gemini C_18_ column using a gradient system of 60:40 to 80:20 CH_3_CN-H_2_O (0.1% formic acid) over 15 min at a flow rate of 21.2 mL/min to yield five subfractions. Subfractions 3 and 4 were subjected to semipreparative reverse-phase HPLC to yield compound **1** (11.7 mg) and compound **2** (26.0 mg), respectively. Subfraction 5 was subjected to semiprepative HPLC to yield both compounds **4** (3.2 mg) and **5** (3.7 mg). UPLC was used to evaluate the purity of compounds **1**–**5** using a gradient solvent system of 15:85 CH_3_CN–H_2_O (0.1% formic acid) to 100% CH_3_CN over 3 min; all compounds were >95% pure (Supporting Information, Figure S28).

#### Embellicine A (**1**)

White amorphous powder; ECD (6.6 × 10^–4^ M, CH_3_CN), λ_max_ (Δε) 302 (−0.90), 289 (0.58), 260 (−4.06), 238 (9.36), and 212 (−17.47) nm; ^1^H NMR (DMSO-*d*_6_, 400 MHz) and ^13^C NMR (DMSO-*d*_6_, 100 MHz) Table S1; HRESIMS *m*/*z* 528.3115 [M + H]^+^ (calcd for C_34_H_42_NO_4_, 528.3113).

#### Embellicine C (**2**)

White amorphous powder; [α]_20_^*D*^ + 44 (*c* 0.1, CH_3_CN); UV (CH_3_CN) λ_max_ (log ε) 274 (3.1), 226 (3.9) nm; ECD (9.2 × 10^–4^ M, CH_3_CN), λ_max_ (Δε) 323 (4.73), 258 (−13.90), 240 (−7.73), and 214 (−30.46) nm; ^1^H NMR (CDCl_3_, 400 MHz) and ^13^C NMR (CDCl_3_, 125 MHz) Tables 1 and 2; HRESIMS *m*/*z* 544.3072 [M + H]^+^ (calcd for C_34_H_42_NO_5_, 544.3063).

#### 2′-O-Acetyl-embellicine C (**3**)

White amorphous powder; [α]_20_^*D*^ −82 (*c* 0.05, CH_3_CN); UV (CH_3_CN) λ_max_ (log ε) 275 (3.1), 227 (4.0) nm; ECD (8.5 × 10^–4^, CH_3_CN), λ_max_ (Δε) 323 (5.50), 258 (−13.00), 248 (−10.91), and 220 (−39.88) nm; ^1^H NMR (CDCl_3_, 500 MHz) and ^13^C NMR (CDCl_3_, 125 MHz) Tables 1 and 2; HRESIMS *m*/*z* 586.3157 [M + H]^+^ (calcd for C_36_H_44_NO_6_, 586.3168).

#### Embellicine D (**4**)

White amorphous powder; [α]_20_^*D*^ + 67 (*c* 0.05, CH_3_CN); UV (CH_3_CN) λ_max_ (log ε) 279 (3.2), 230 (4.0) nm; ECD (9.2 × 10^–4^ M, CH_3_CN), λ_max_ (Δε) 316 (14.84), 276 (−7.70), 255 (−6.59), and 219 (−17.68) nm; ^1^H NMR (CDCl_3_, 400 MHz) and ^13^C NMR (CDCl_3_, 100 MHz) Tables 1 and 2; HRESIMS *m*/*z* 544.3038 [M + H]^+^ (calcd for C_34_H_42_NO_5_, 544.3063).

#### Embellicine E (**5**)

White amorphous powder; [α]_20_^*D*^ −320 (*c* 0.035, CH_3_CN); UV (CH_3_CN) λ_max_ (log ε) 267 (3.6), 222 (3.8) nm; ECD (6.3 × 10^–4^ M, CH_3_CN), λ_max_ (Δε) 289 (2.23), 260 (−15.30), 233 (−4.20), and 221 (−6.31) nm; ^1^H NMR (acetone-*d*_6_, 400 MHz) and ^13^C NMR (acetone-*d*_6_, 125 MHz) Tables 1 and 2; HRESIMS *m*/*z* 560.3354 [M + H]^+^ (calcd for C_35_H_46_NO_5_, 560.3376).

### Acetylation of Embellicine C (2)

Pyridine was used to dissolve 5 mg of **2**, which was placed in an ice bath before gradually adding 100 μL of acetic anhydride. The reaction was left stirring at room temperature for 5 h and then evaporated to dryness. The reaction mixture was subjected to semipreparative HPLC over a Phenomenex Synergi C_12_ column using a gradient system of 40:60 to 90:10 CH_3_CN-H_2_O (0.1% formic acid) over 15 min at a flow rate of 4.7 mL/min to afford 2 mg of the acetylated product, 2′-*O*-acetyl-embellicine C (**3**).

### Cytotoxicity Assay

To evaluate the cytotoxic activity of compounds **1**–**5**, human melanoma cancer cells MDA-MB-435, human breast cancer cells MDA-MB-231, and human ovarian cancer cells OVCAR3 were purchased from the American Type Culture Collection. The cell lines were propagated at 37 °C in 5% CO_2_ in RPMI 1640 medium, supplemented with fetal bovine serum (10%), penicillin (100 units/mL), and streptomycin (100 μg/mL). Cells in log phase growth were harvested by trypsinization followed by two washings to remove all traces of enzyme. A total of 5000 cells was seeded per well of a 96-well clear, flat-bottom plate (Microtest 96, Falcon) and incubated overnight (37 °C in 5% CO_2_). Samples dissolved in DMSO were then diluted and added to the appropriate wells. The cells were incubated in the presence of a test substance for 72 h at 37 °C and evaluated for viability with a commercial absorbance assay (CellTiter-Blue Cell Viability Assay, Promega Corp) that measured viable cells. IC_50_ values are expressed in μM relative to the solvent (DMSO) control; taxol (paclitaxel) was used as a positive control.

### Computational Methods

Molecular Merck force field (MMFF) calculations were carried out with Spartan’10 (Wave function Inc.) to obtain the minimum energy conformers of **2** and **5**. These calculations resulted in one major conformer, each, for both **2** and **5**. For the ECD prediction, the resulting conformers were optimized using a time-dependent DFT (TDDFT) method at the B3LYP/6-311G+(2d,p) level in CH_3_CN. Then, the TDDFT method at the B3LYP/6-31G+(d) level of theory was employed for ECD calculations. All calculations were performed via the Gaussian’09 program package (Gaussian Inc.),^57,58^ while the calculated ECD spectra were plotted using Specdis software and compared to the experimental ECD data.^59^

## Data Availability

The NMR data for **1**–**5** were deposited in the NP-MRD (https://np-mrd.org/) under accession numbers NP0331524, NP0331528, NP0331526, NP0331525, and NP0331527, respectively.

## Supporting Information Available

HRESIMS data, ^1^H and ^13^C NMR of compounds **1**–**5**, 2D NMR data (COSY, HSQC, HMBC, and NOESY) for the new compounds (**2**–**5**), experimental ECD spectra of compounds **1**–**5**, and UPLC purity chromatograms of compounds **1**–**5** (PDF)

## Dedication

Dedicated to Dr. Mary J. Garson, The University of Queensland, for her pioneering work on bioactive natural products.
