# Supplementary material for: Embellicines C-E: Macrocyclic Alkaloids with a Cyclopenta[b]fluorene Ring System from the Fungus Sarocladium sp
Source: J Nat Prod. 2023 Mar 8;86(3):596–603. doi: 10.1021/acs.jnatprod.2c01048 (PMC10043936; doi:10.1021/acs.jnatprod.2c01048)
Supplement: Supplementary file 1 — np2c01048_si_001.pdf [file np2c01048_si_001.pdf]

Supporting Information

Embellicines C-E: Macrocyclic Alkaloids with a Cyclopenta[b]fluorene Ring System from the Fungus *Sarocladium* sp.

Zeinab Y. Al Subeh, [#,†] Laura Flores-Bocanegra,[†] Huzefa A. Raja, [†] Joanna E. Burdette,[#] Cedric J. Pearce,[§] Nicholas H. Oberlies[*,†]

[#]Department of Medicinal Chemistry and Pharmacognosy, Faculty of Pharmacy, Jordan University of Science and Technology, Irbid 22110, Jordan.

[†]Department of Chemistry and Biochemistry, University of North Carolina at Greensboro, Greensboro, North Carolina 27402, United States.

[§]Mycosynthetix, Inc., Hillsborough, North Carolina 27278, United States.

[#]Department of Pharmaceutical Sciences, University of Illinois at Chicago, Chicago, Illinois 60612, United States

[*]Email: nicholas_oberlies@uncg.edu

Supporting Information Content

Figure S1. (+)-HRESIMS spectra of compounds **1–5**.

Figure S2. ^{1}H and ^{13}C NMR spectra of compoud **1** [400 MHz for ^{1}H and 100 MHz for ^{13}C, DMSO-*d*$_6$].

Table S1. Collected NMR data of embellicine A (**1)** in DMSO-*d*$_6$ as compared to those reported in the literature.

Figure S3. ^{1}H and ^{13}C NMR spectra of **2** [400 MHz for ^{1}H and 100 MHz for ^{13}C, $CDCl_3$].

Figure S4. Edited HSQC NMR spectrum of **2** [500 MHz, $CDCl_3$].

Figure S5. COSY NMR spectrum of **2** [500 MHz, $CDCl_3$].

Figure S6. HMBC NMR spectrum of **2** [500 MHz, $CDCl_3$].

Figure S7. NOESY NMR spectrum of **2** [400 MHz, $CDCl_3$].

Table S2. Collected NMR data of **2** in $CDCl_3$ compared to phomapyrrolidone C as reported by Wijeratne et. al.

Table S3. Collected NMR data of **2** in CD_3OD as compared to ascomylactam C as reported by Chen et. al.

Figure S8. NOESY correlations for the tetracyclic cyclopenta[b]fluorene (6/5/6/5) ring system in embellicine C (**2**) shown in the 3D structure.

Figure S9. ECD spectrum of embellicine C (**2**) as compared to the reported ECD spectra for its diastereomers: phomapyrrolidone C and ascomylactam C.

Figure S10. ^{1}H and ^{13}C NMR spectra of **3** [500 MHz for ^{1}H and 125 MHz for ^{13}C, $CDCl_3$].

Figure S11. Edited HSQC NMR spectrum of **3** [500 MHz, $CDCl_3$].

Figure S12. COSY NMR spectrum of **3** [500 MHz, $CDCl_3$].

Figure S13. HMBC NMR spectrum of **3** [500 MHz, $CDCl_3$].

Figure S14. NOESY NMR spectrum of **3** [400 MHz, $CDCl_3$].

Figure S15. ^{1}H and ^{13}C NMR spectra of **4** [400 MHz for ^{1}H and 100 MHz for ^{13}C, $CDCl_3$].

Figure S16. Edited HSQC NMR spectrum of **4** [400 MHz, $CDCl_3$].

Figure S17. COSY NMR spectrum of **4** [400 MHz, $CDCl_3$].

Figure S18. HMBC NMR spectrum of **4** [400 MHz, $CDCl_3$].

Figure S19. NOESY NMR spectrum of **4** [400 MHz, $CDCl_3$].

Figure S20. Experimental ECD spectra of compounds **2–4** in CH_3CN.

Figure S21. UPLC chromatograms of **2** (A) and **4** (B) collected four months after their first isolation, showing interconversion between the two compounds.

Figure S22. ^{1}H and ^{13}C NMR spectra of **5** [400 MHz for ^{1}H and 100 MHz for ^{13}C, acetone-*d*$_6$].

Figure S23. Edited HSQC NMR spectrum of **5** [500 MHz, acetone-*d*$_6$].

Figure S24. COSY NMR spectrum of **5** [500 MHz, acetone-d_6].

Figure S25. HMBC NMR spectrum of **5** [500 MHz, acetone-d_6].

Figure S26. NOESY NMR spectrum of **5** [500 MHz, acetone-d_6].

Figure S27. Experimental ECD spectra of compounds **1** and **5** in CH_3CN.

Figure S28. UPLC chromatograms of compounds **1–5** (λ 210 nm), demonstrating > 95% purity.

Figure S29. Proposed biosynthesis of embellicines with cyclopenta[b]fluorene (6/5/6/5) ring system.

Figure S30. *Sarocladium* sp. (MSX6737). **a.** Vegetative hyphae, **b.** Cylindrical phialide arising from vegetative hypha, **c.** Conidia arranged in slimy heads, **d.** Conidia in slimy heads arising from ropes of vegetative hyhae.

Figure S31. Molecular phylogenetics analysis of *Sarocladium*.

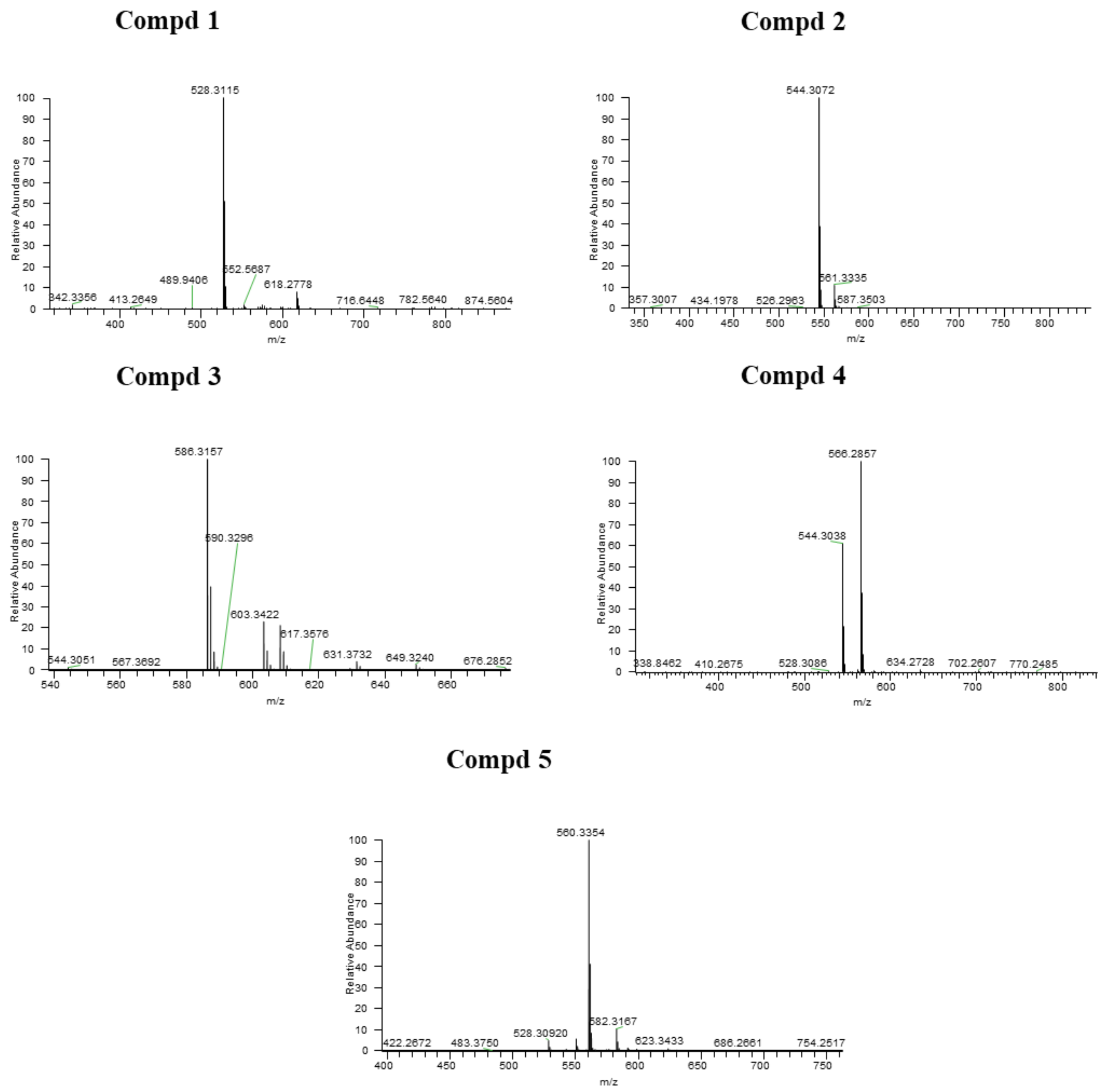

Figure S1. (+)-HRESIMS spectra of compounds **1–5**.

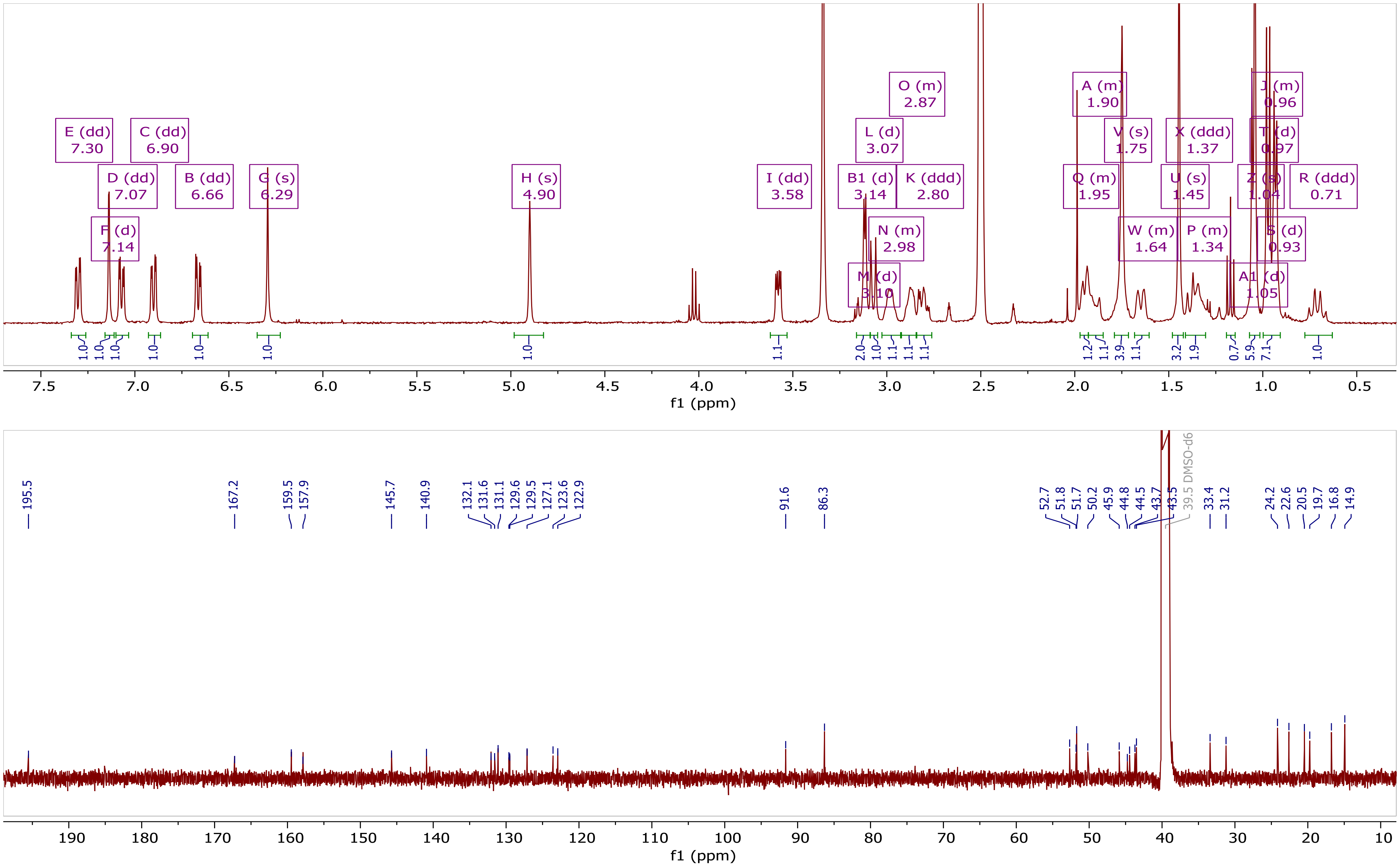

Figure S2. ^{1}H and ^{13}C NMR spectra of compoud **1** [400 MHz for ^{1}H and 100 MHz for ^{13}C, DMSO-d_6].

Table S1. Collected NMR data of embellicine A (**1**) in DMSO-d_6 as compared to those reported in the literature.[3]

	Collected for embellicine A (**1**)		Reported for embellicine A (**1**)	
position	δ_C, type	δ_H (J, Hz)	δ_C, type	δ_H (J, Hz)
1	44.5, CH	2.98, m	44.4, CH	2.99, m
2	140.9, C		140.9, C	
3	129.6, CH	4.90, br s	129.5, CH	4.90, s
4	51.8, C		51.8, C	
5	145.7, C		145.7, C	
6	127.1, C		127.0, C	
7	43.5, CH	2.87, m	43.5, CH	2.88, m
8	45.9, CH	1.90, m	45.8, CH	1.91, m
9	38.8, CH_2	1.95, m	38.8, CH_2	1.95, m
		1.37, ddd (11.5, 11.5, 11.5)		1.38, ddd (11.4, 11.4, 11.4)
10	33.4, CH	1.34, m	33.3, CH	1.34, m
11	43.7, CH_2	1.64, m	43.8, CH_2	1.66, m
		0.71, ddd (11.9, 11.9, 11.9)		0.71, ddd (12.0, 12.0, 12.0)
12	31.2, CH	1.76, m	31.1, CH	1.77, m
13	51.7, CH	0.96, m	51.6, CH	0.96, ddd (3.3, 6.8, 8.1)
14	91.6, CH	3.58, dd (7.4, 3.3)	91.6, CH	3.59, dd (3.5, 7.4)
15	50.2, CH	2.80, ddd (10.5, 8.6, 3.3)	50.1, CH	2.81, ddd (3.6, 8.6, 10.6)
16	52.7, CH	3.07, d (10.5)	52.6, CH	3.08, d (10.8)
17	195.5, C		195.4, C	
18	131.1, C		131.1, C	
19	167.2, C		167.2, C	
20	16.8, CH_3	0.97, d (6.8)	16.7, CH_3	0.98, d (6.8)
21	14.9, CH_3	1.45, s	14.8, CH_3	1.45, s
22	24.2, CH_3	1.04, s	24.1, CH_3	1.04, s
23	19.7, CH_3	1.75, s	19.6, CH_3	1.75, s
24	22.6, CH_3	0.93, d (6.3)	22.5, CH_3	0.94, d (6.2)
25	20.5, CH_3	1.05, d (7.3)	20.4, CH_3	1.05, d (7.3)
1'	157.9, CH	7.14, d (1.6)	157.7, CH	7.11, d (1.7)
2'	86.3, C		86.2, C	
3'	44.8, CH_2	3.14, d (12.3)	44.7, CH_2	3.14, d (12.2)
		3.10, d (12.3)		3.10, d (12.2)
4'	132.1, C		131.9, C	
5'	129.5, CH	6.90, dd (8.4, 2.0)	129.4, CH	6.91, dd (8.4, 2.2)
6'	122.9, CH	6.66, dd (8.4, 2.4)	122.8, CH	6.67, dd (8.3, 2.5)
7'	159.5, C		159.4, C	
8'	123.6, CH	7.07, dd (8.2, 2.4)	123.5, CH	7.07, dd (8.2, 2.5)
9'	131.6, CH	7.30, dd (8.2, 2.0)	131.5, CH	7.30, dd (8.2, 2.2)

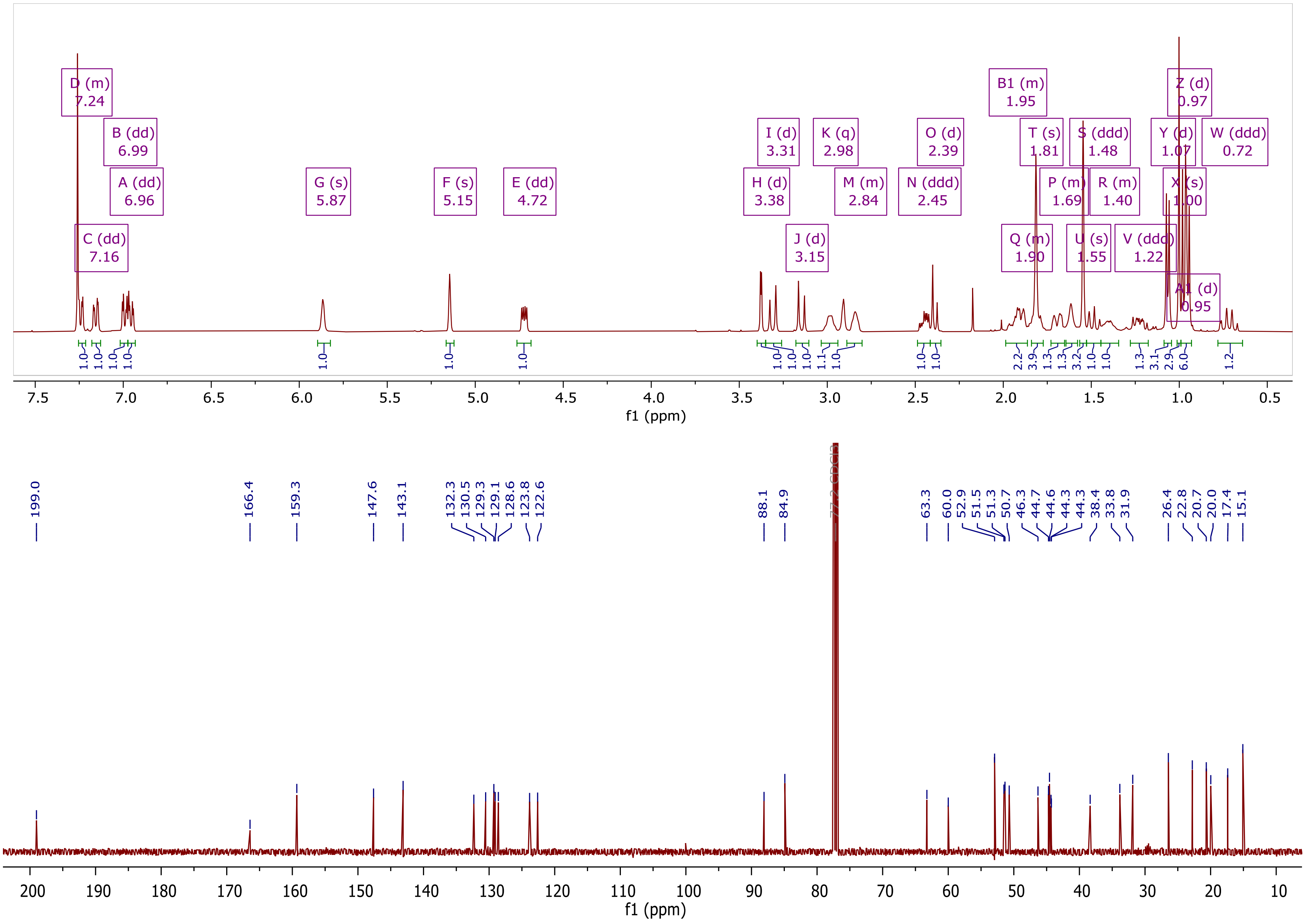

Figure S3. ^{1}H and ^{13}C NMR spectra of **2** [400 MHz for ^{1}H and 100 MHz for ^{13}C, $CDCl_3$].

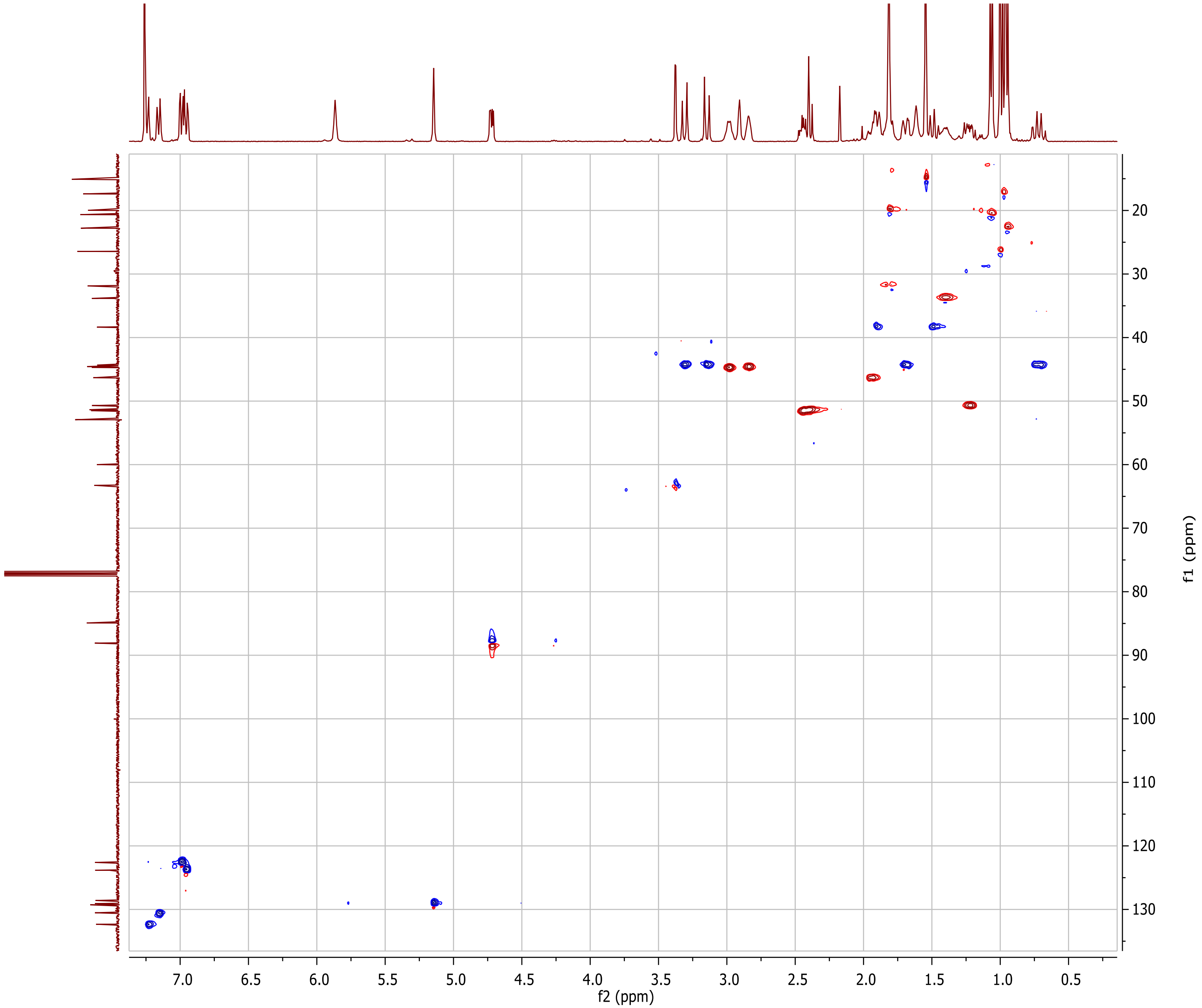

Figure S4. Edited HSQC NMR spectrum of **2** [500 MHz, $CDCl_3$].

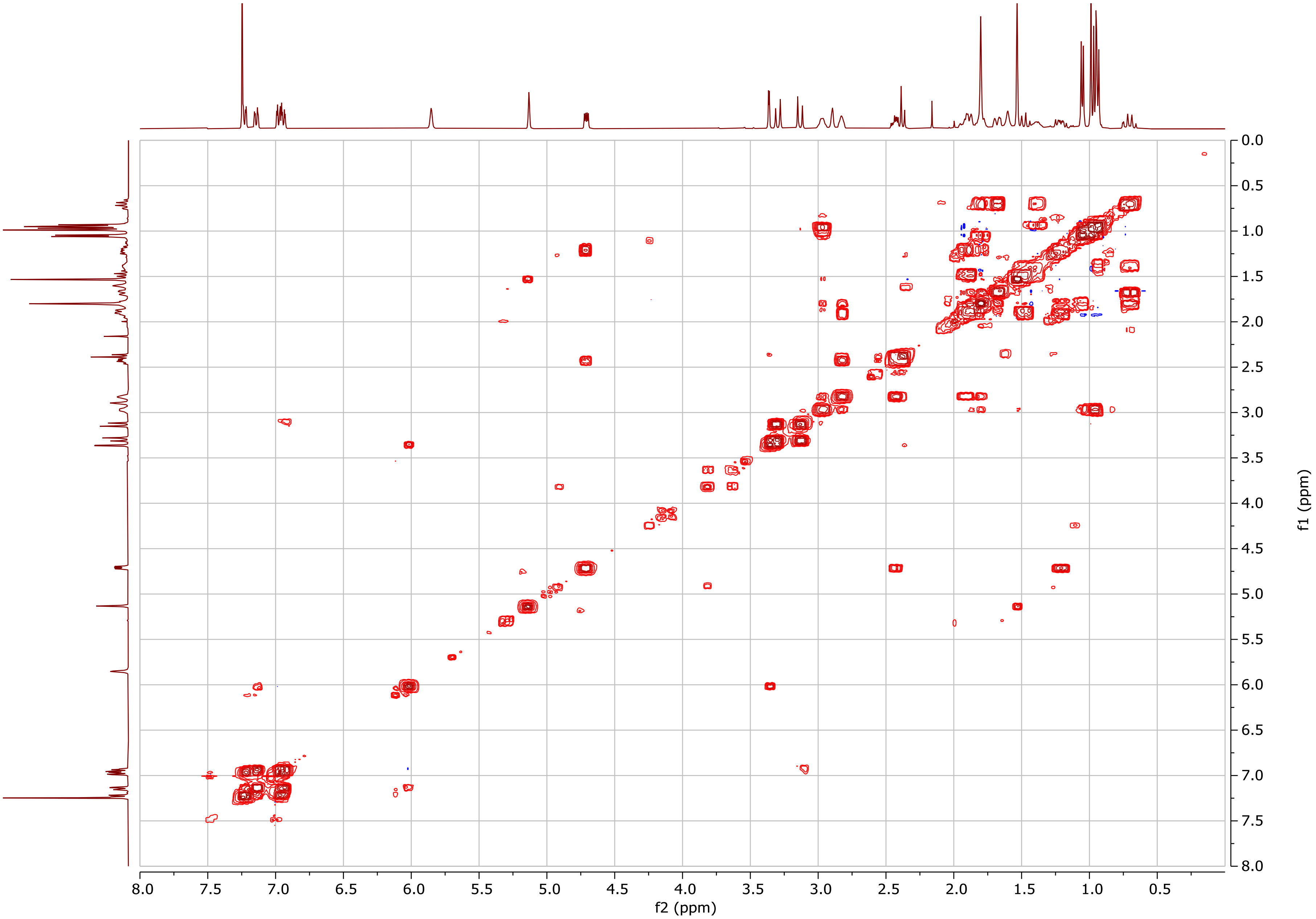

Figure S5. COSY NMR spectrum of **2** [500 MHz, $CDCl_3$].

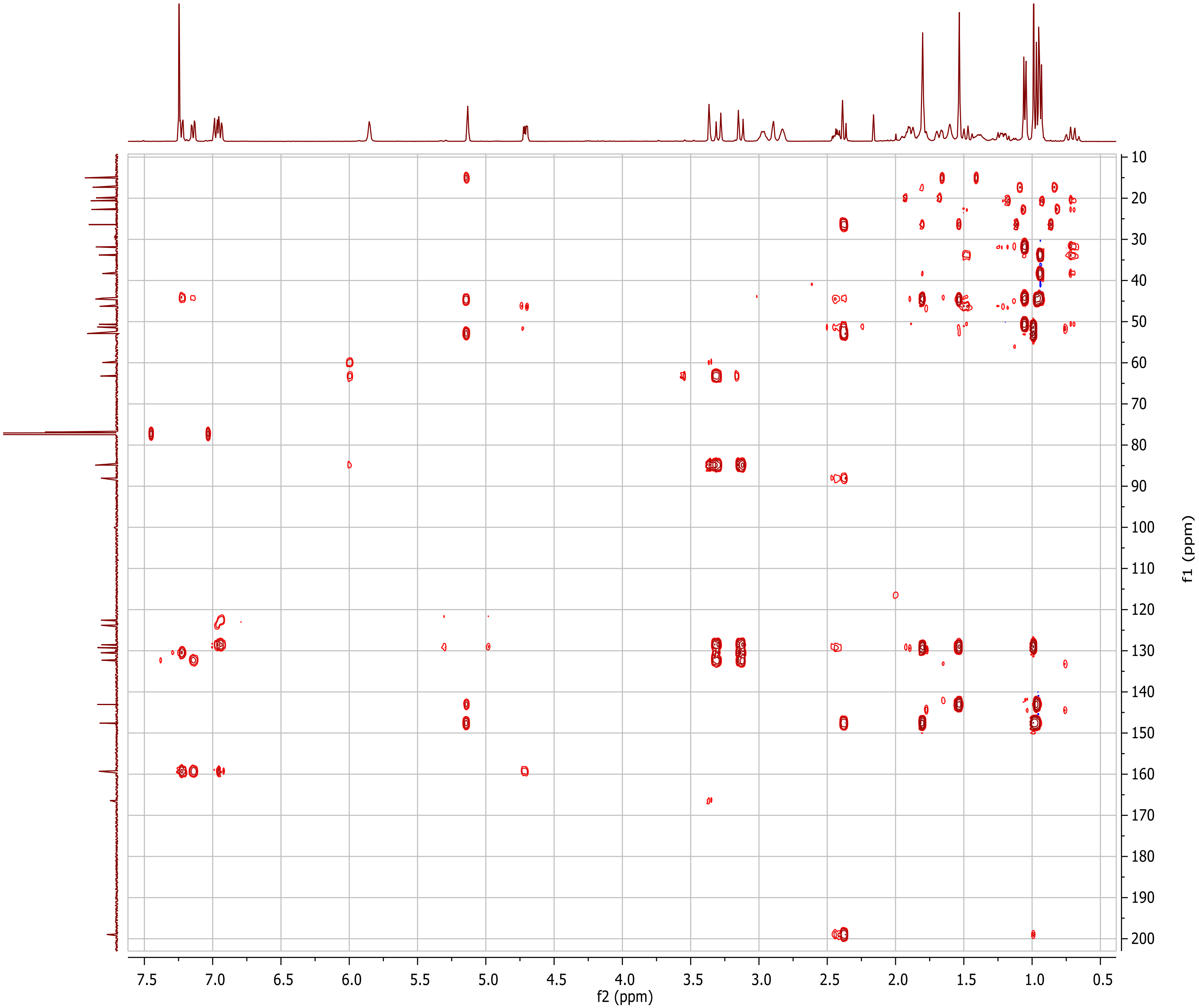

Figure S6. HMBC NMR spectrum of **2** [500 MHz, $CDCl_3$].

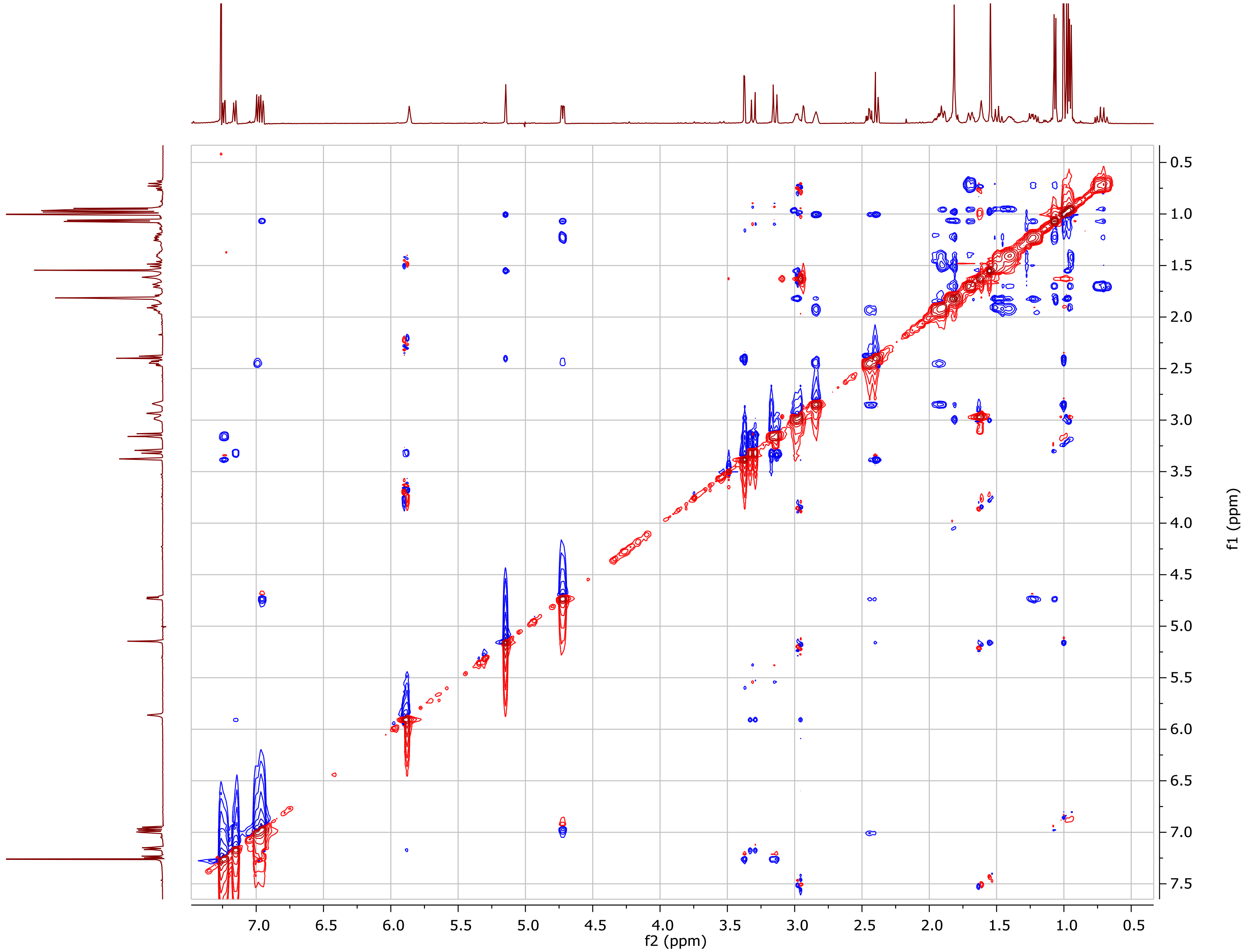

Figure S7. NOESY NMR spectrum of **2** [400 MHz, $CDCl_3$].

\

Table S2. Collected NMR data of **2** in $CDCl_3$ compared to phomapyrrolidone C as reported by Wijeratne et. al.[1, 2]

	**2**	Phomapyrrolidone C
position	δ_H (J, Hz)	δ_H (J, Hz)
1	2.98, q (7.4)	2.95, q (7.2)
3	5.15, s	5.28, t (1.6)
7	2.84, m	1.93, t (11.2)
8	1.95, m	1.56, m
9a	1.90 (m)	2.09, brd (3.2)
9b	1.48, q (11.9)	0.73, m
10	1.40 (m)	1.54, m
11a	1.69 (m)	1.81, m
11b	0.72, q (12.0)	0.74, m
12	1.82, m	1.84, m
13	1.22, ddd (13.6, 10.4, 7.9)	1.11, m
14	4.72, dd (7.7, 3.7)	4.26, dd (8.0, 5.6)
15	2.45, ddd (10.5, 6.9, 3.8)	1.78, m
16	2.39, d (10.2)	2.04, d (10.0)
20	0.97, d (7.0)	1.02, d (7.2)
21	1.55, br s	1.62, s
22	1.00, s	0.74, s
23	1.81, s	1.76, s
24	0.95, d (6.5)	0.92, d (6.4)
25	1.07, d (6.4)	1.13, d (6.0)
1'	3.38, d (2.4)	3.53, m
3'a	3.31, d (13.6)	3.35, d (14.0)
3'b	3.15, d (13.6)	3.15, d (14.0)
5'	7.16, dd (8.4, 2.0)	7.19, dd (8.0, 2.0)
6'	6.96, dd (8.4, 2.3)	7.08, dd (8.0, 2.0)
8'	6.99, dd (8.1, 2.3)	6.98, dd (8.0, 2.0)
9'	7.24, dd (8.1, 2.0)	7.20, dd (8.0, 2.0)
19-NH	5.87, brs	6.60, brs

Table S3. Collected NMR data of **2** in CD_3OD as compared to ascomylactam C as reported by Chen et. al.[2]

	**2**	Ascomylactam C
position	δ_H (J, Hz)	δ_H (J, Hz)
1	3.00, q (6.9)	2.75, q (7.3)
3	5.24, t (1.4)	5.31, s
7	2.89, m	2.09, t (11.5)
8	1.99, m	1.62, m
9a	1.95, m	2.18, d (12.6)
9b	1.54, m	0.80, m
10	1.40, m	1.55, m
11a	1.71, m	1.82, m
11b	0.74, ddd (12.1, 12.1, 12.1)	0.71, m
12	1.88, m	1.86, m
13	1.20, ddd (13.5, 10.5, 7.7)	1.26, m
14	4.65, dd (7.7, 2.6)	4.82, m
15	2.58, m	2.0, dt (6.3, 12.5)
16	2.58, m	3.76, d (6.2)
20	0.99, d (6.9)	0.98, d (4.5)
21	1.53, s	1.53, s
22	1.01, s	1.27, s
23	1.83, s	1.80, s
24	0.98, d (6.5)	0.97, d (3.9)
25	1.11, d (6.4)	1.10, d (6.1)
1'	3.62, s	3.56, brs
3'a	3.24, d (13.3)	3.05, brs
3'b	3.09, d (13.3)	
5'	7.10, dd (8.5, 2.1)	7.07, dd (2.0, 8.3)
6'	6.82, dd (8.5, 2.4)	7.19, dd (2.3, 8.3)
8'	7.02, dd (8.1, 2.4)	6.60, dd (2.3, 8.2)
9'	7.34, dd (8.1, 2.1)	7.10, dd (2.0, 8.2)

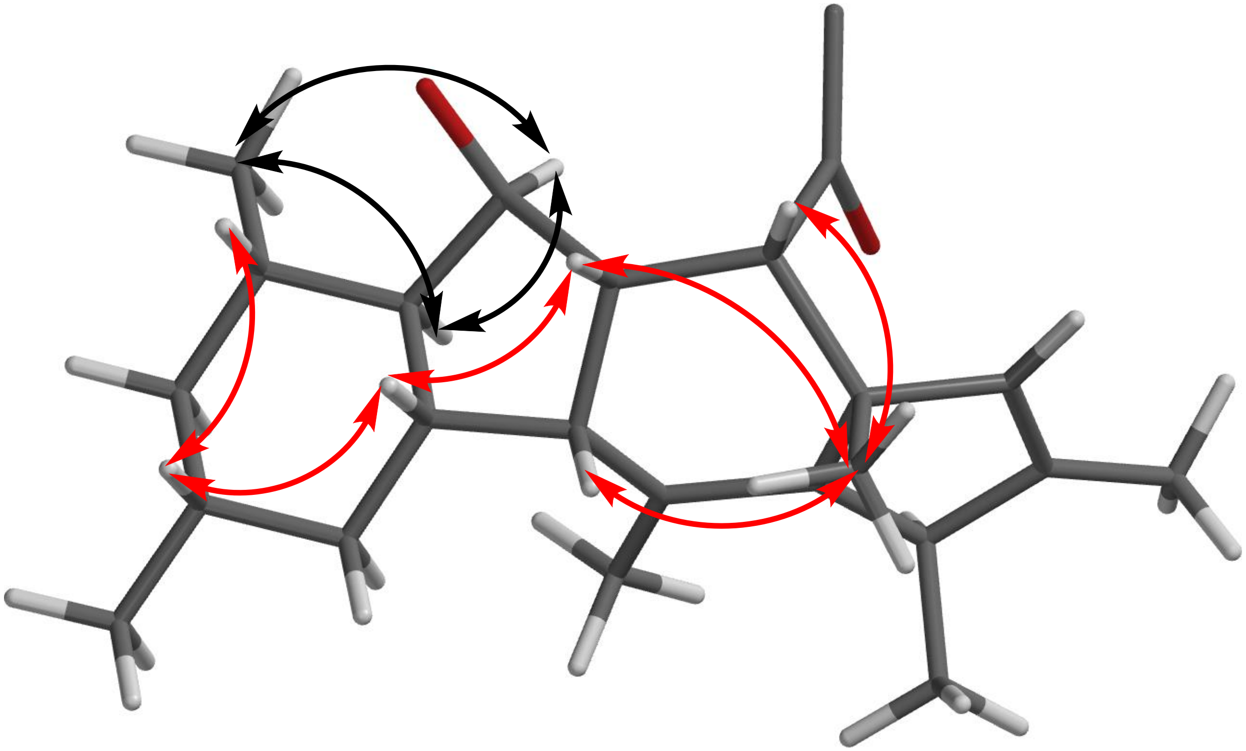

Figure S8. NOESY correlations for the tetracyclic cyclopenta[b]fluorene (6/5/6/5) ring system in embellicine C (**2**) shown in the 3D structure. The red vs black arrows indicate NOESY correlations on opposite faces of the molecule.

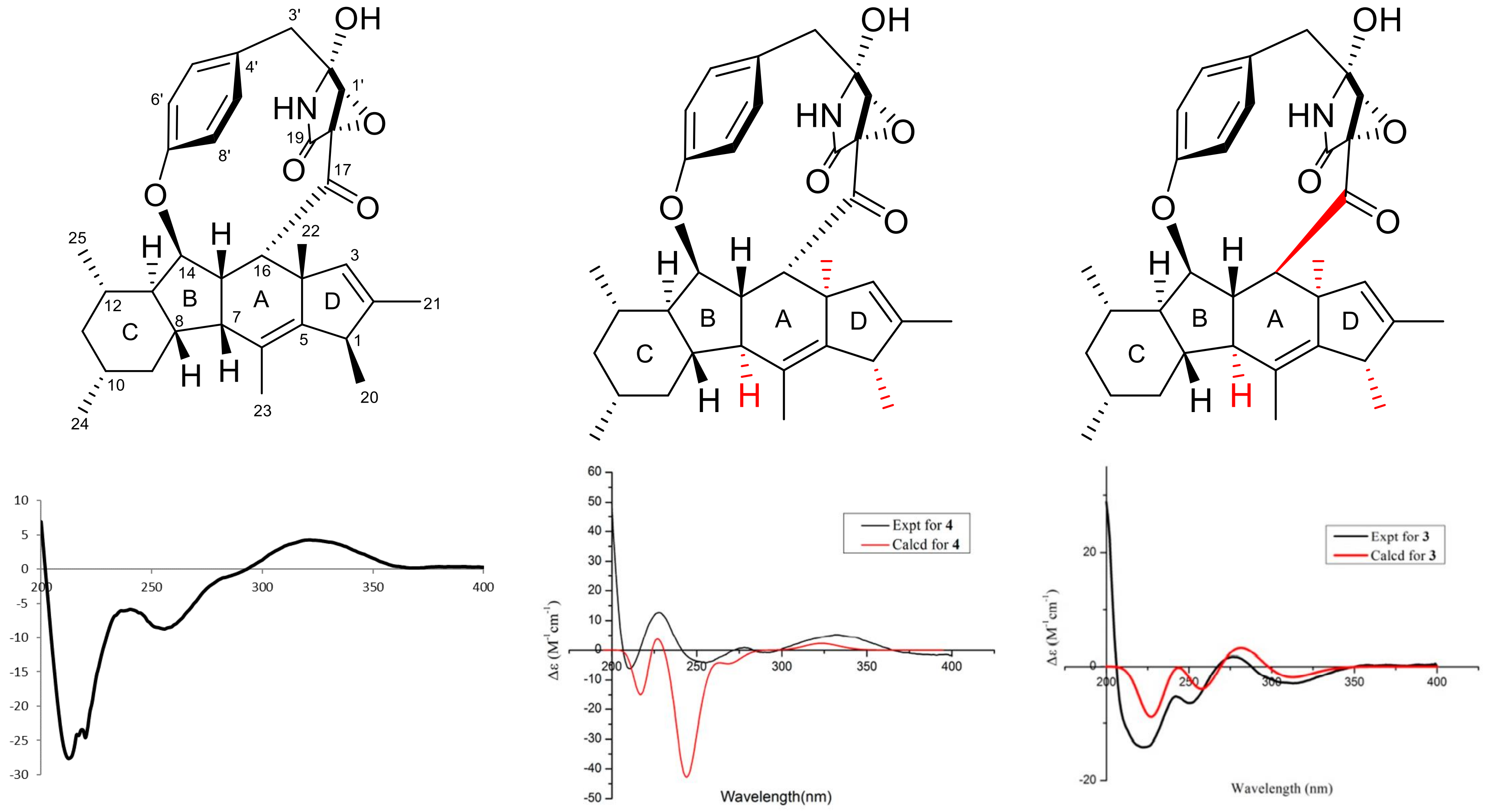

Embellicine C (**2**) Phomapyrrolidone C Ascomylactam C

Figure S9. ECD spectrum of embellicine C (**2**) as compared to the reported ECD spectra for its diastereomers: phomapyrrolidone C and ascomylactam C.[2] The compound numbers in the phomapyrrolidone C and ascomylacam C spectra correlate to how they are reported in their respective publications. In the top structural drawings, the red colored bonds indicate differences from **2**.

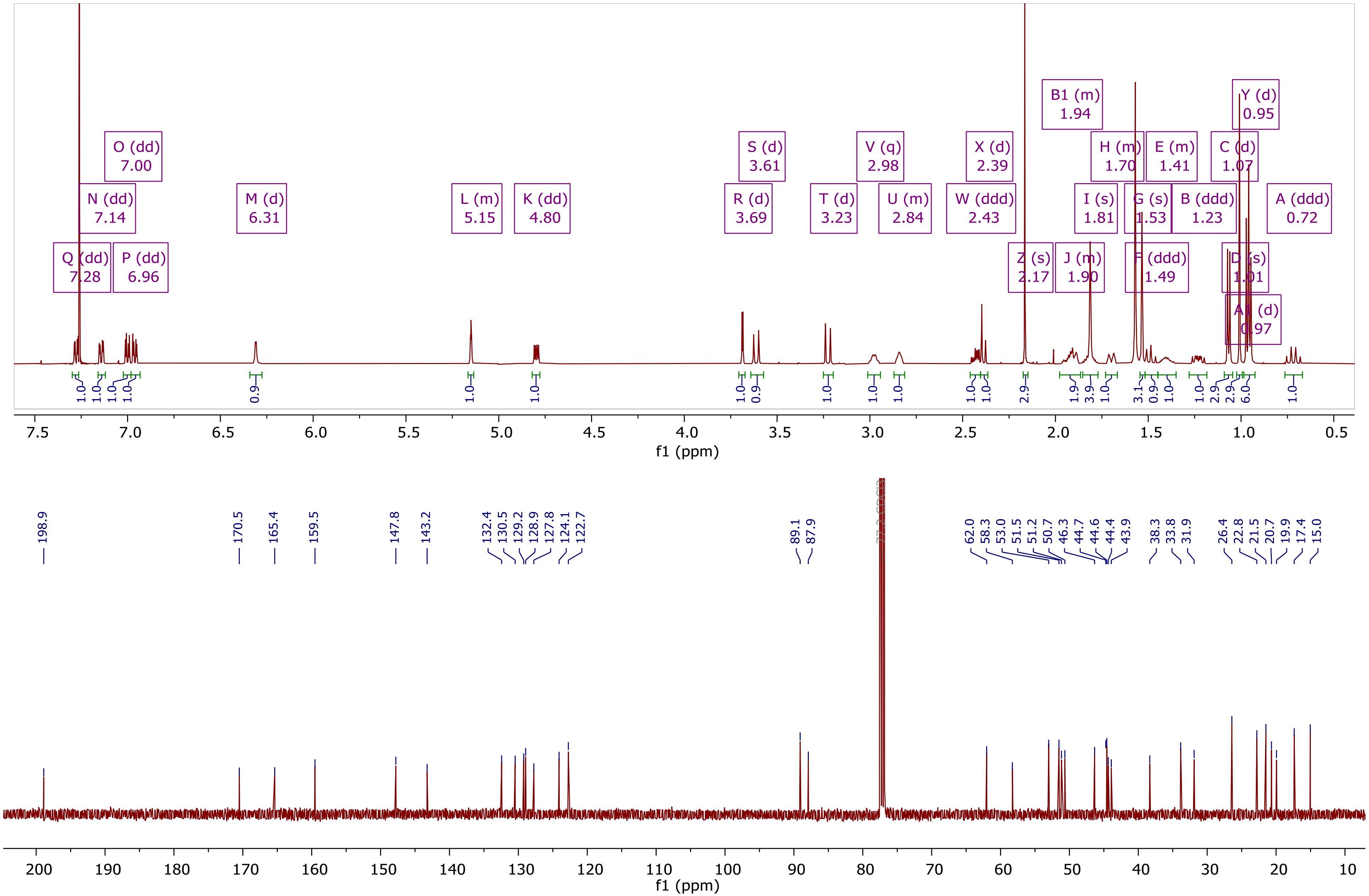

Figure S10. ^{1}H and ^{13}C NMR spectra of **3** [500 MHz for ^{1}H and 125 MHz for ^{13}C, $CDCl_3$].

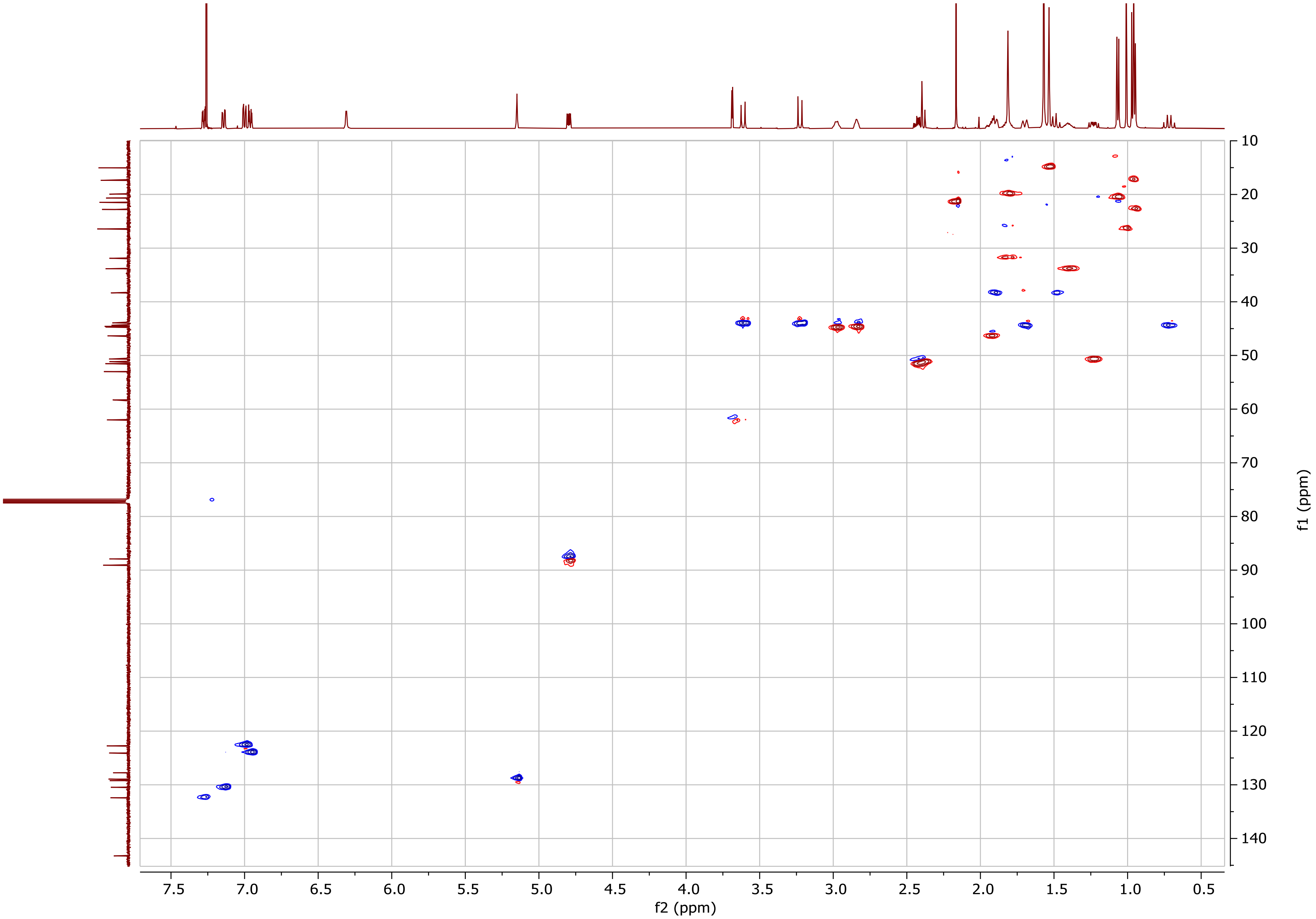

Figure S11. Edited HSQC NMR spectrum of **3** [500 MHz, $CDCl_3$].

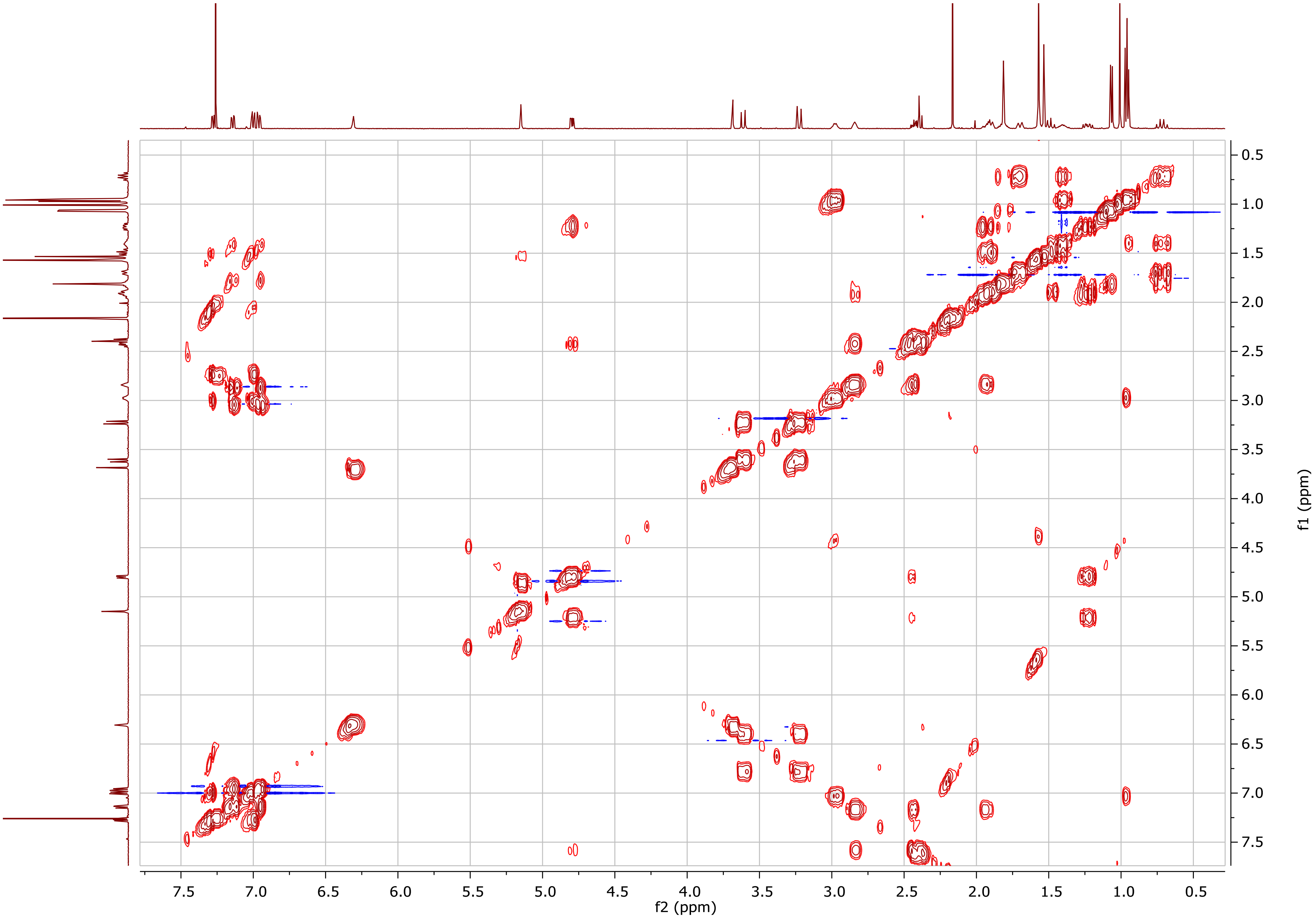

Figure S12. COSY NMR spectrum of **3** [500 MHz, $CDCl_3$].

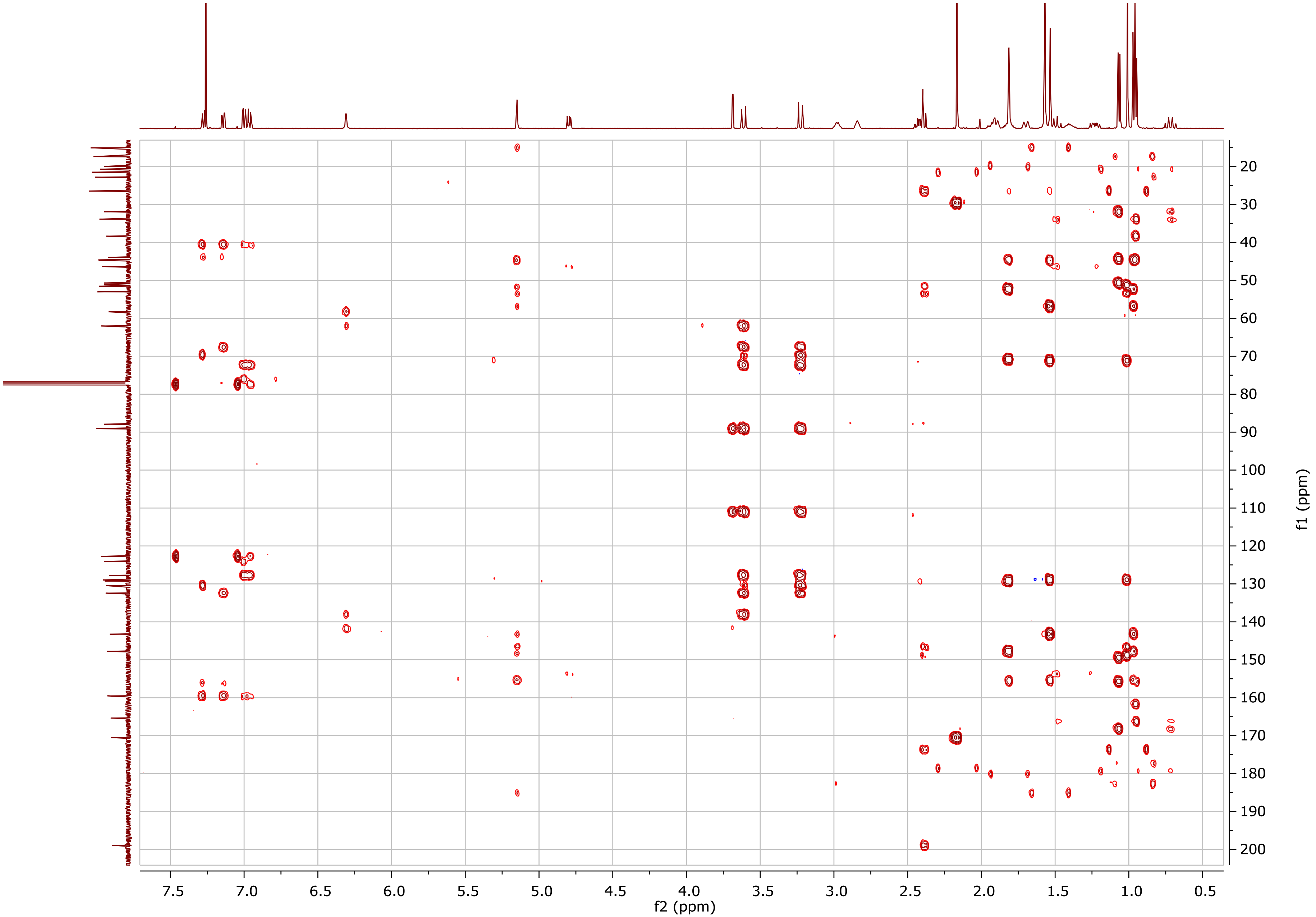

Figure S13. HMBC NMR spectrum of **3** [500 MHz, $CDCl_3$].

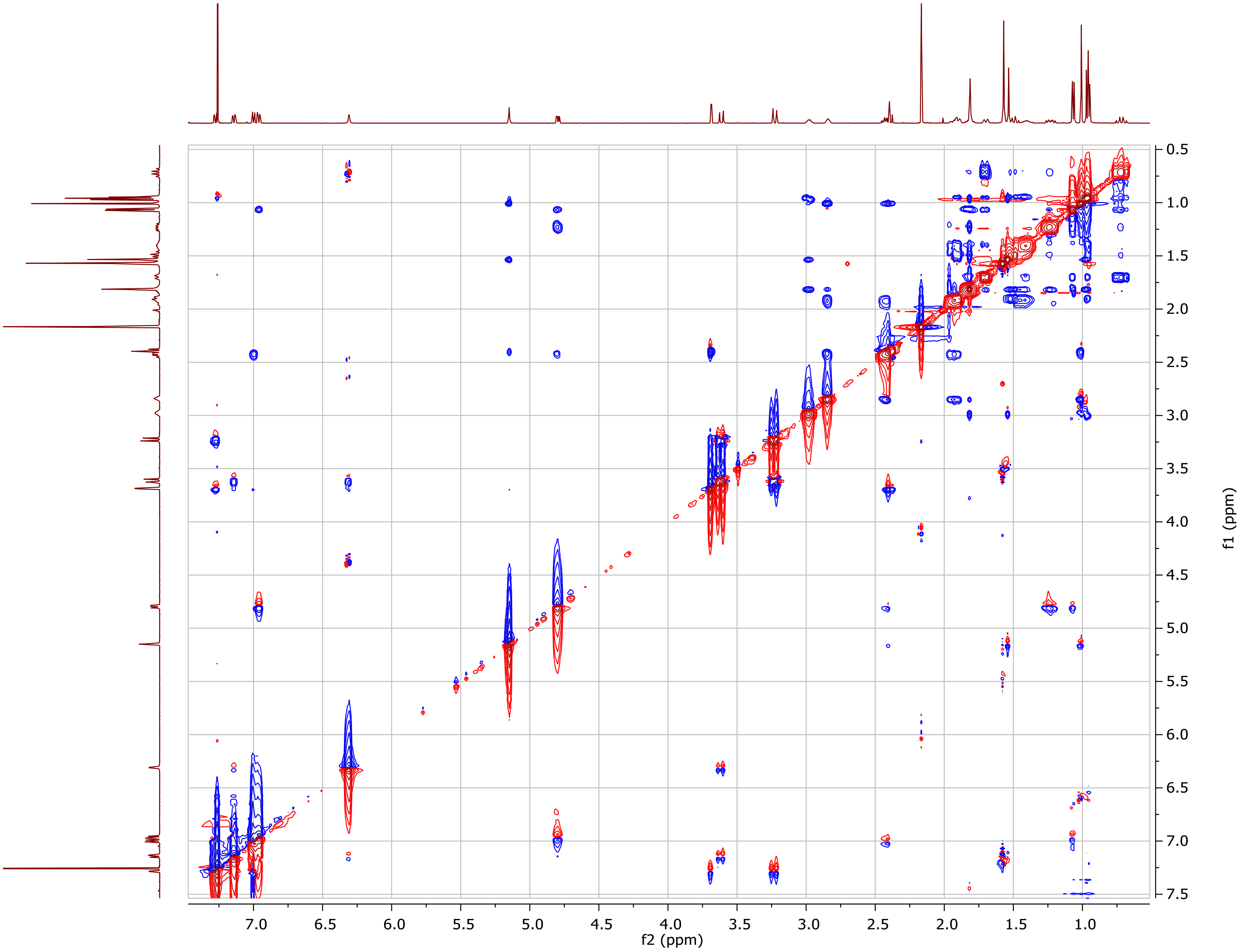

Figure S14. NOESY NMR spectrum of **3** [400 MHz, $CDCl_3$].

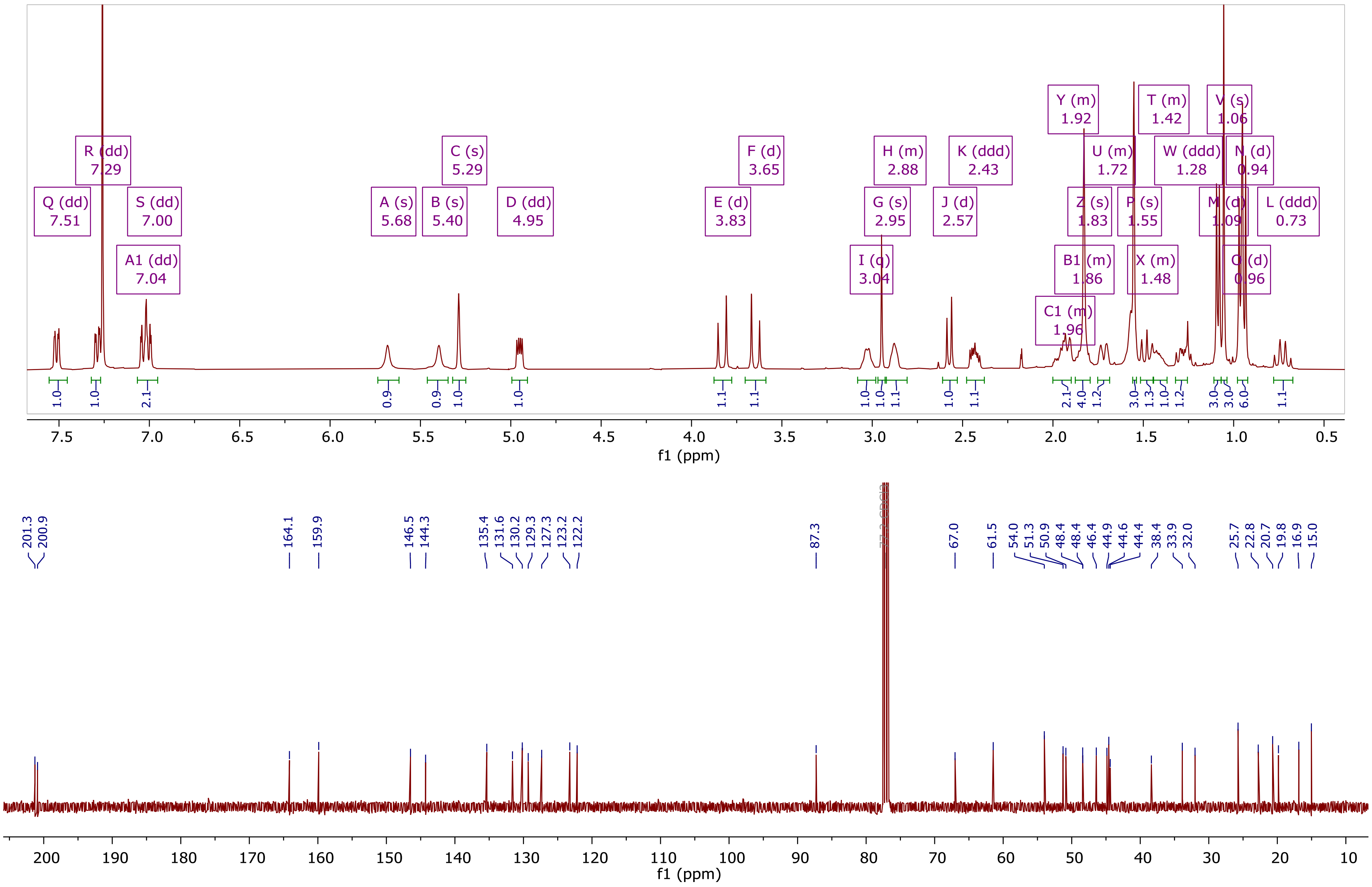

Figure S15. ^{1}H and ^{13}C NMR spectra of **4** [400 MHz for ^{1}H and 100 MHz for ^{13}C, $CDCl_3$].

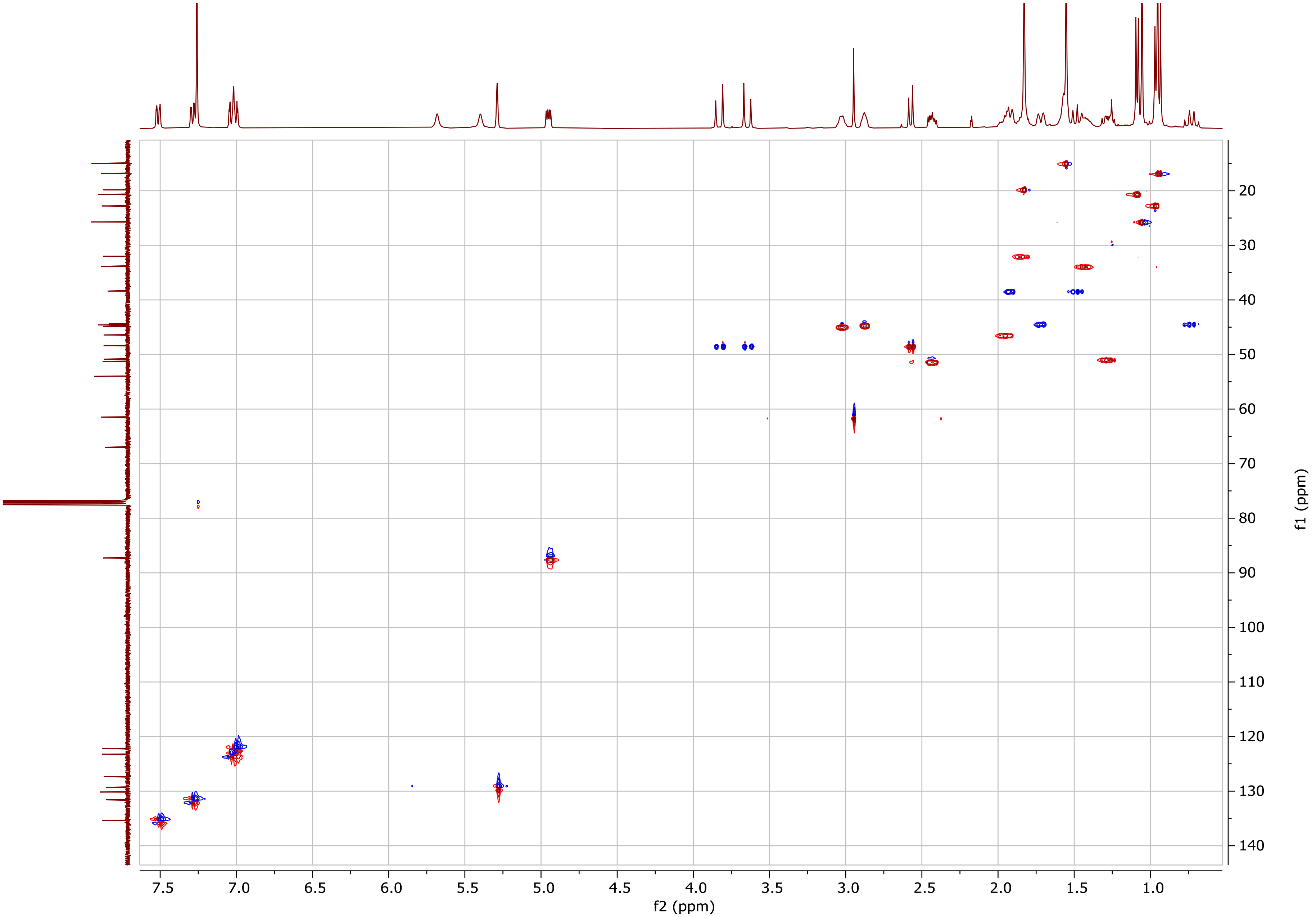

Figure S16. Edited HSQC NMR spectrum of **4** [400 MHz, $CDCl_3$].

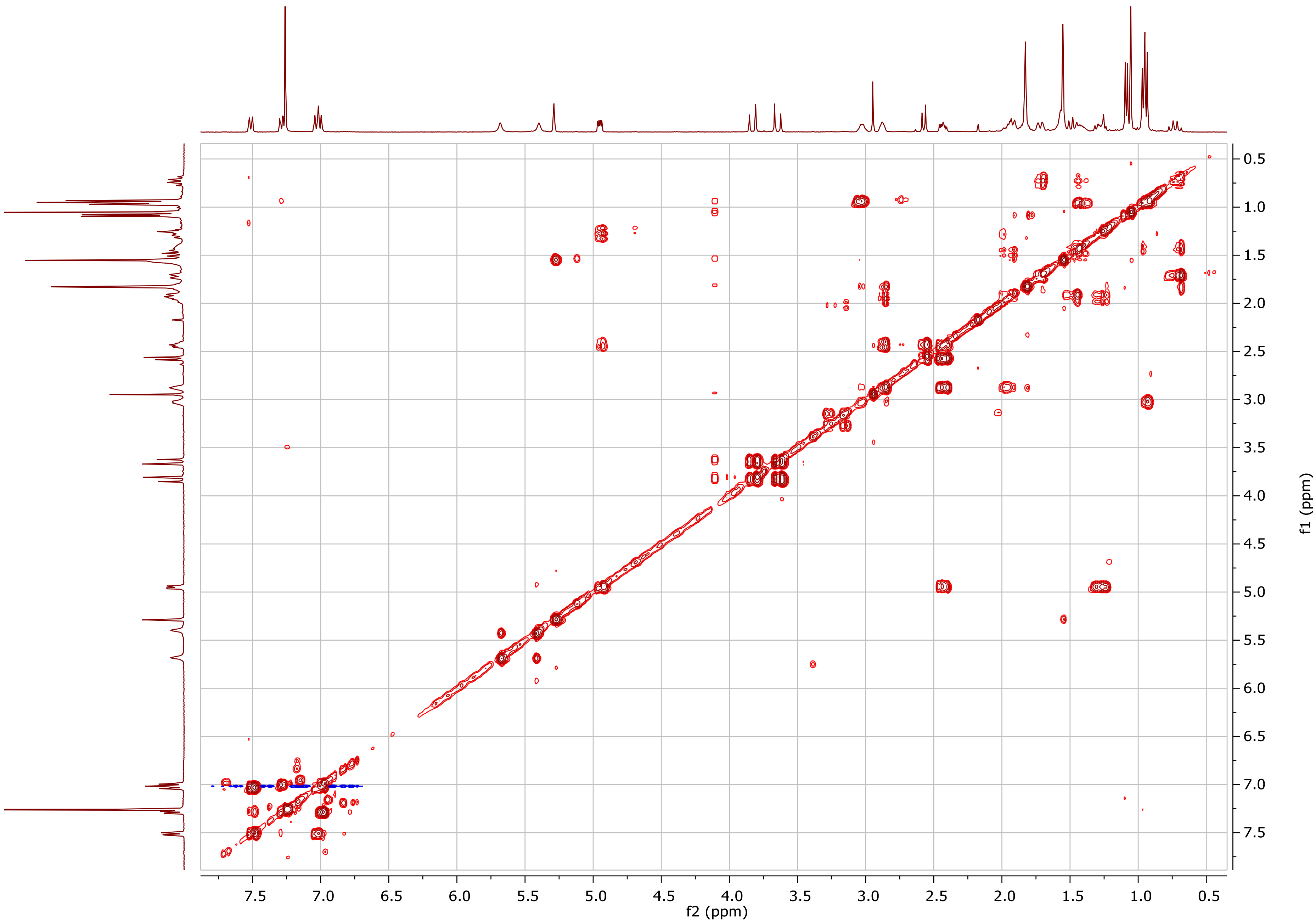

Figure S17. COSY NMR spectrum of **4** [400 MHz, $CDCl_3$].

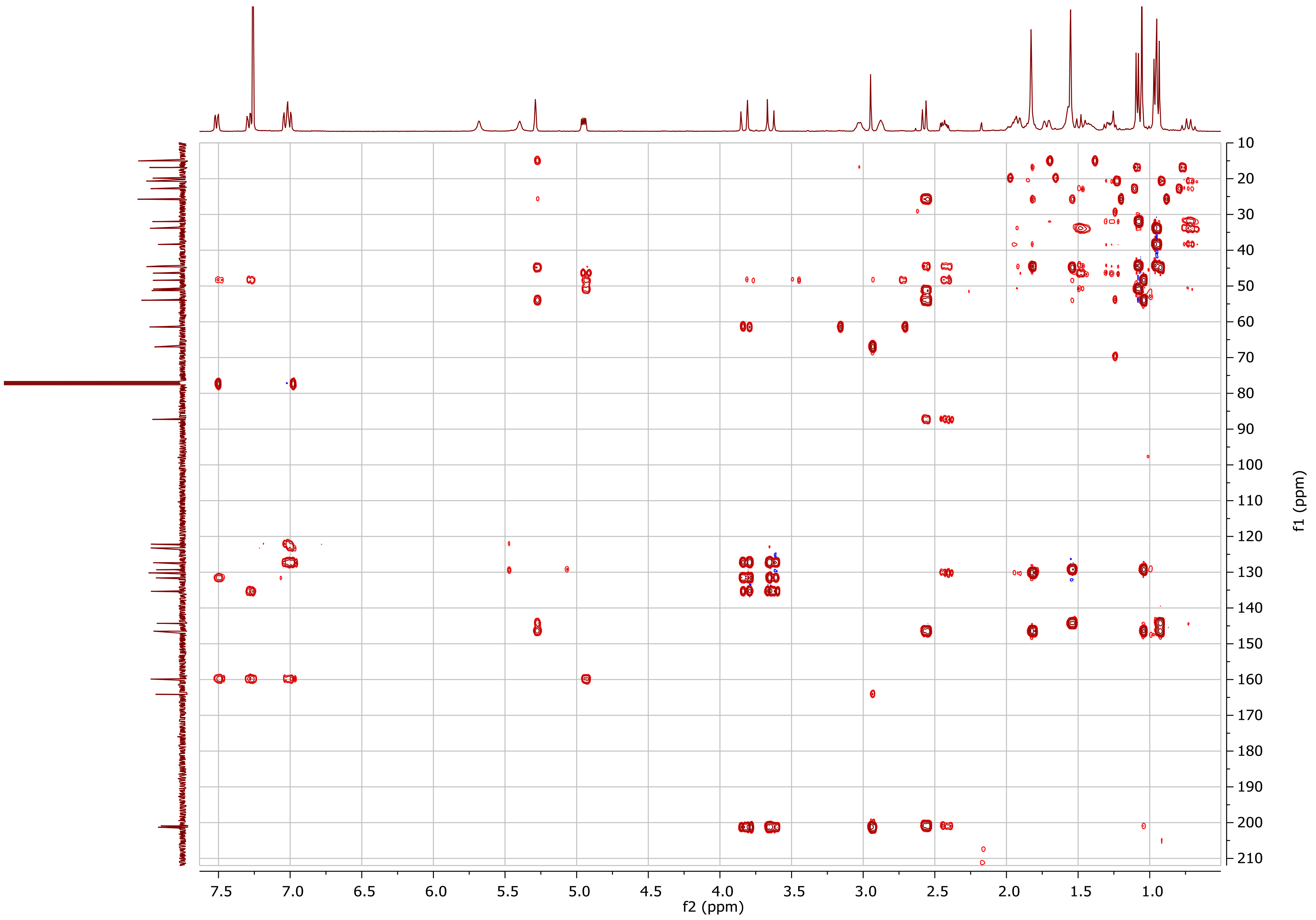

Figure S18. HMBC NMR spectrum of **4** [400 MHz, $CDCl_3$].

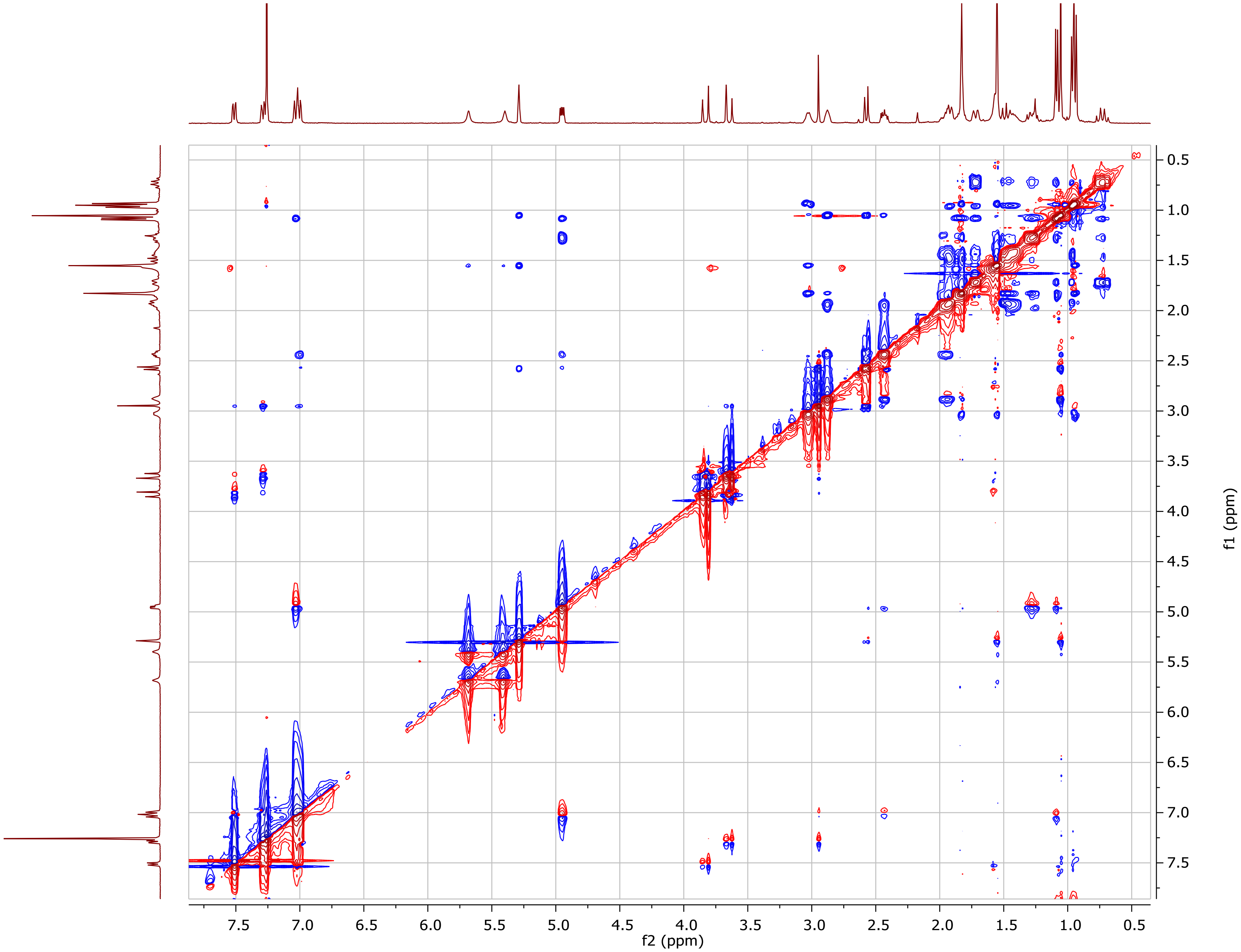

Figure S19. NOESY NMR spectrum of **4** [400 MHz, $CDCl_3$].

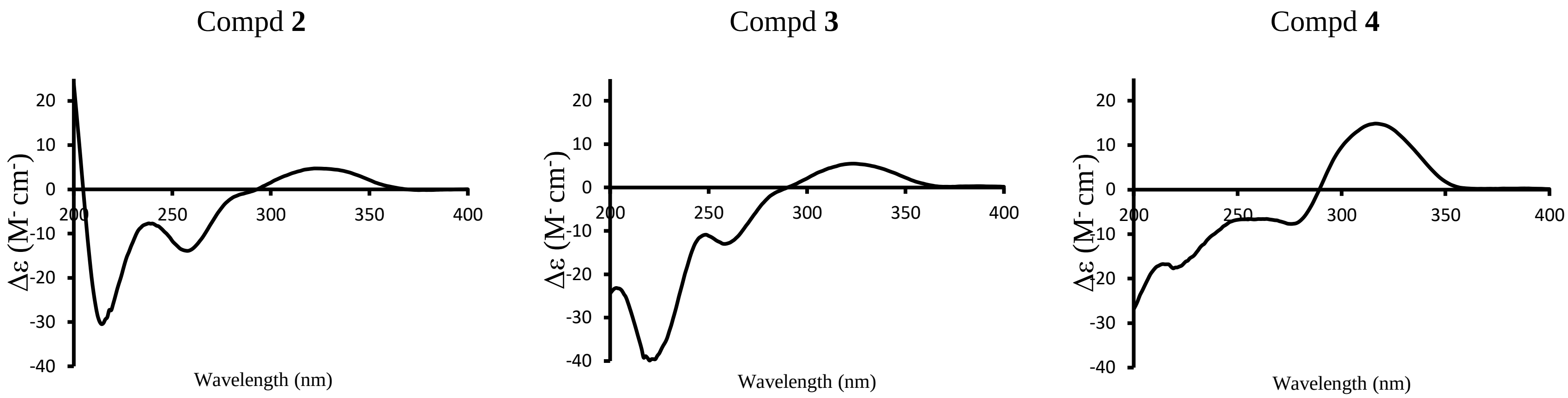

Figure S20. Experimental ECD spectra of compounds **2–4** in CH_3CN.

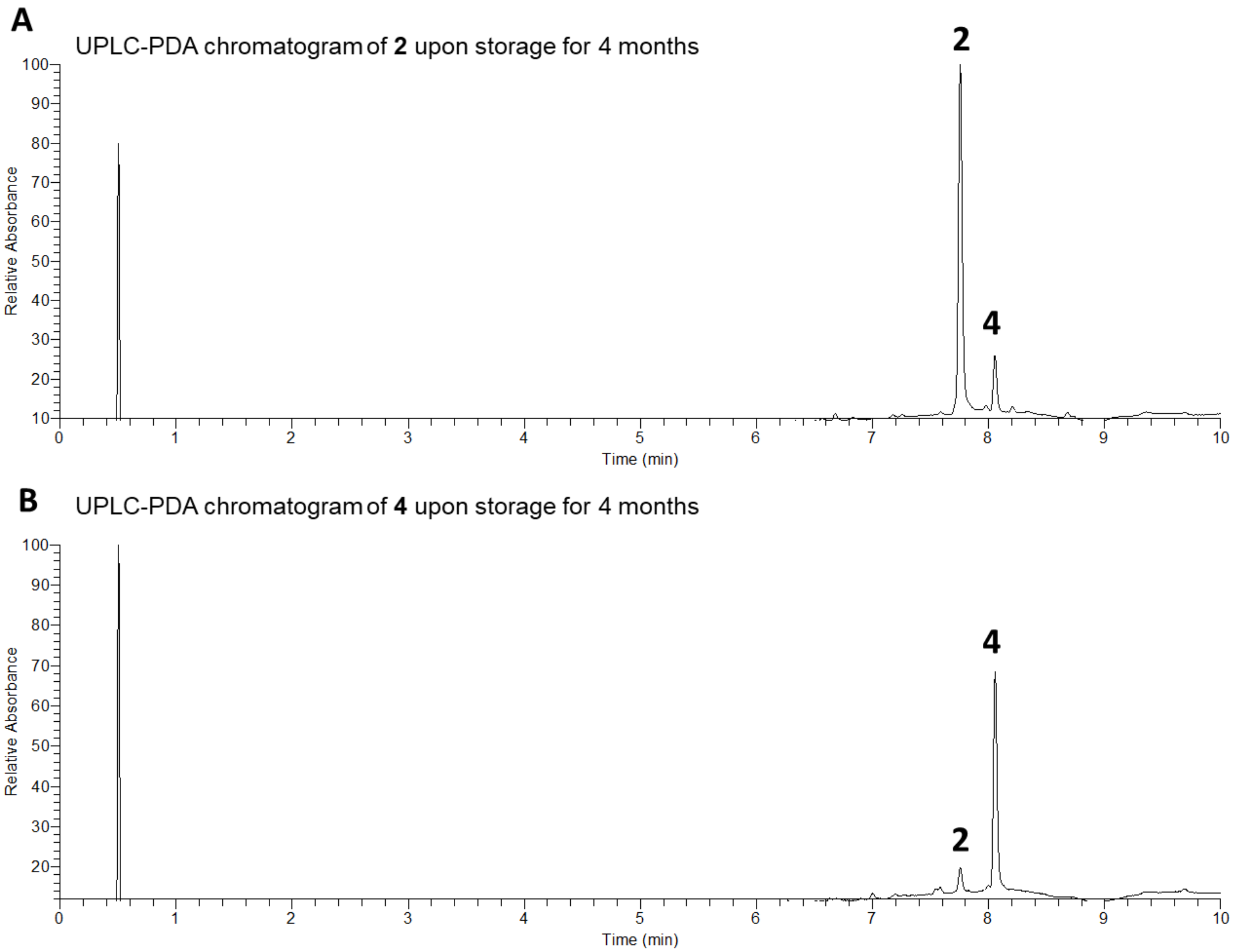

Figure S21. UPLC chromatograms of **2** (A) and **4** (B) collected four months after their first isolation, showing interconversion between the two compounds.

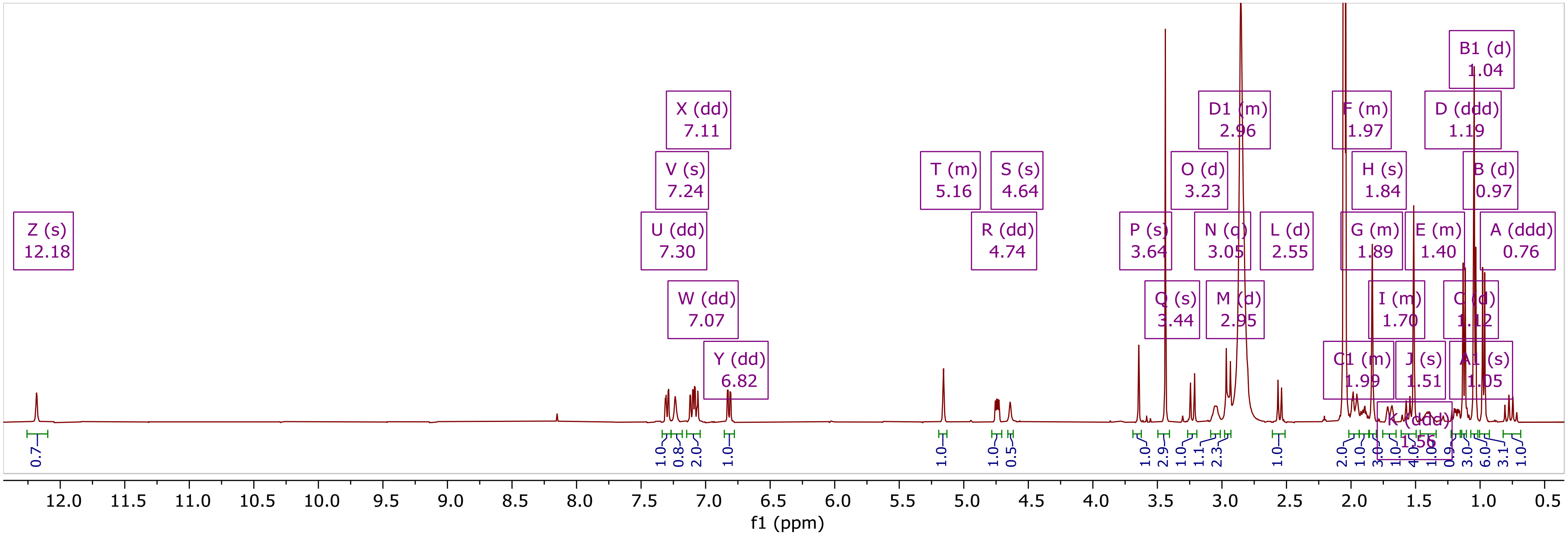

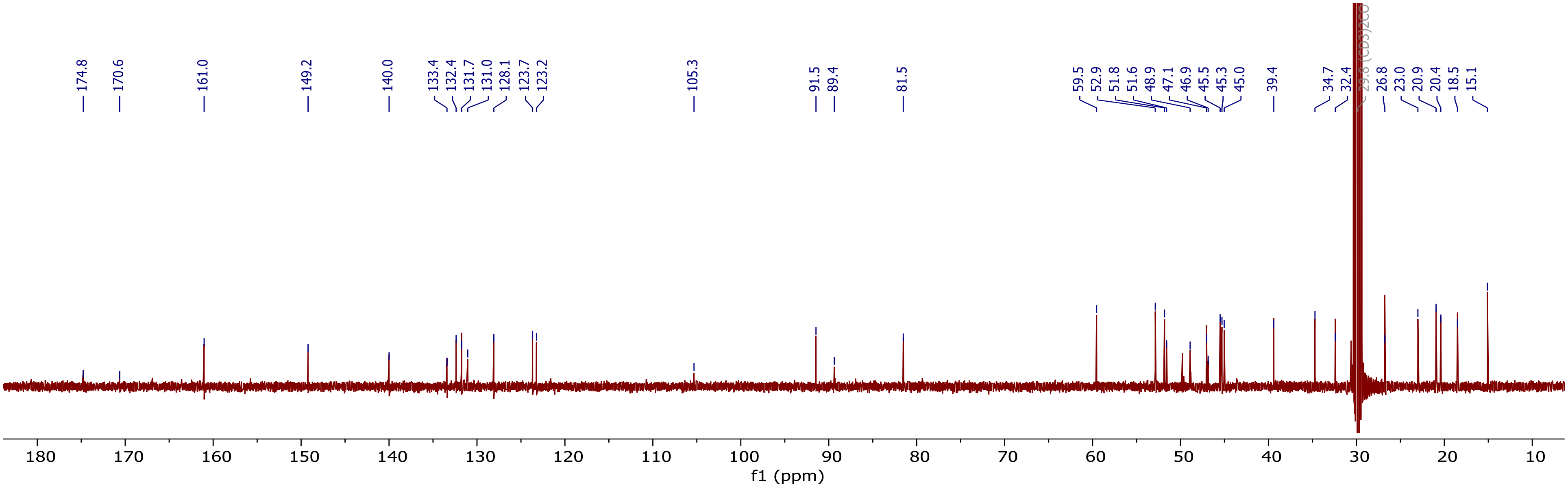

Figure S22. ^{1}H and ^{13}C NMR spectra of **5** [400 MHz for ^{1}H and 100 MHz for ^{13}C, acetone-d_6].

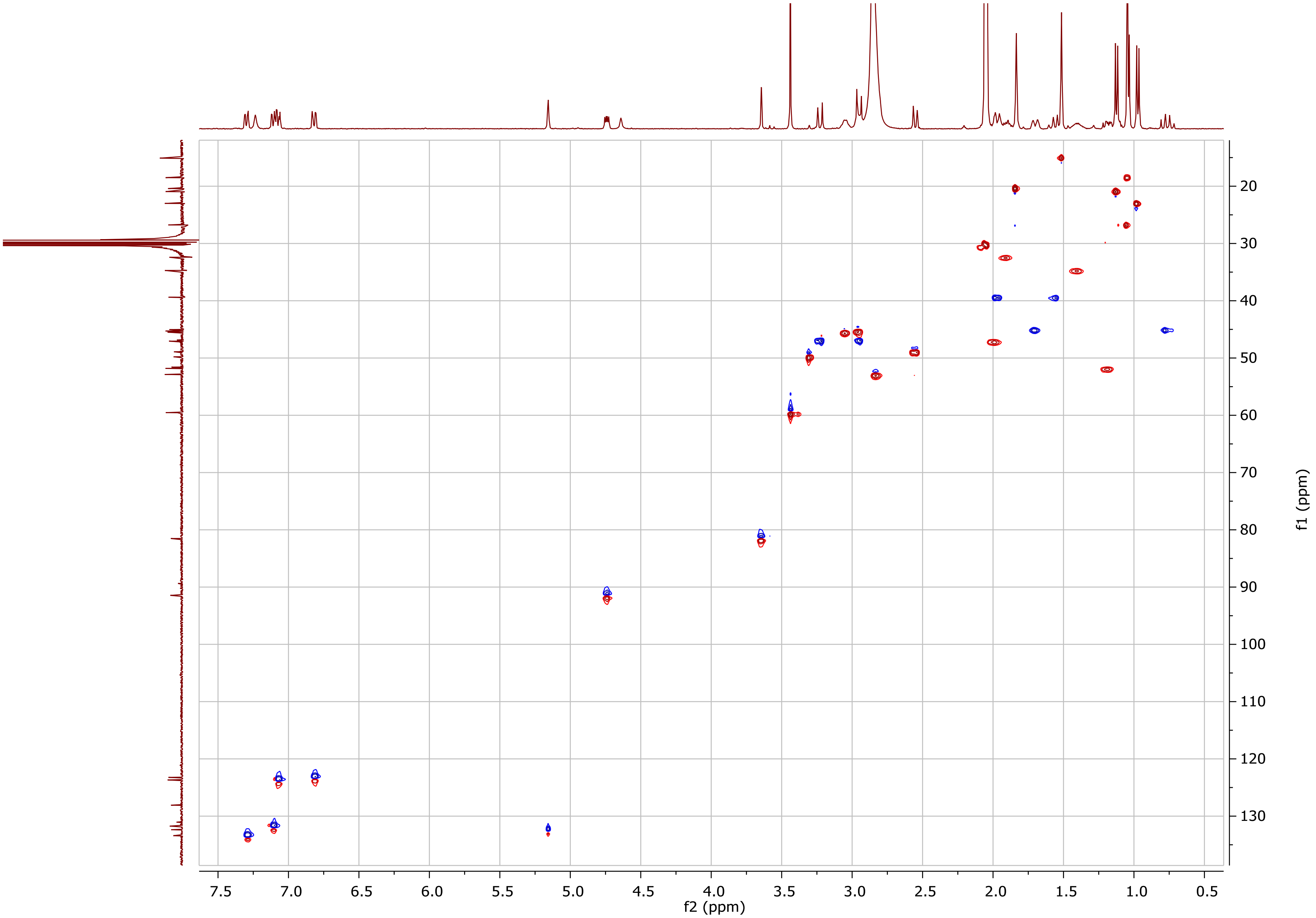

Figure S23. Edited HSQC NMR spectrum of **5** [500 MHz, acetone-d_6].

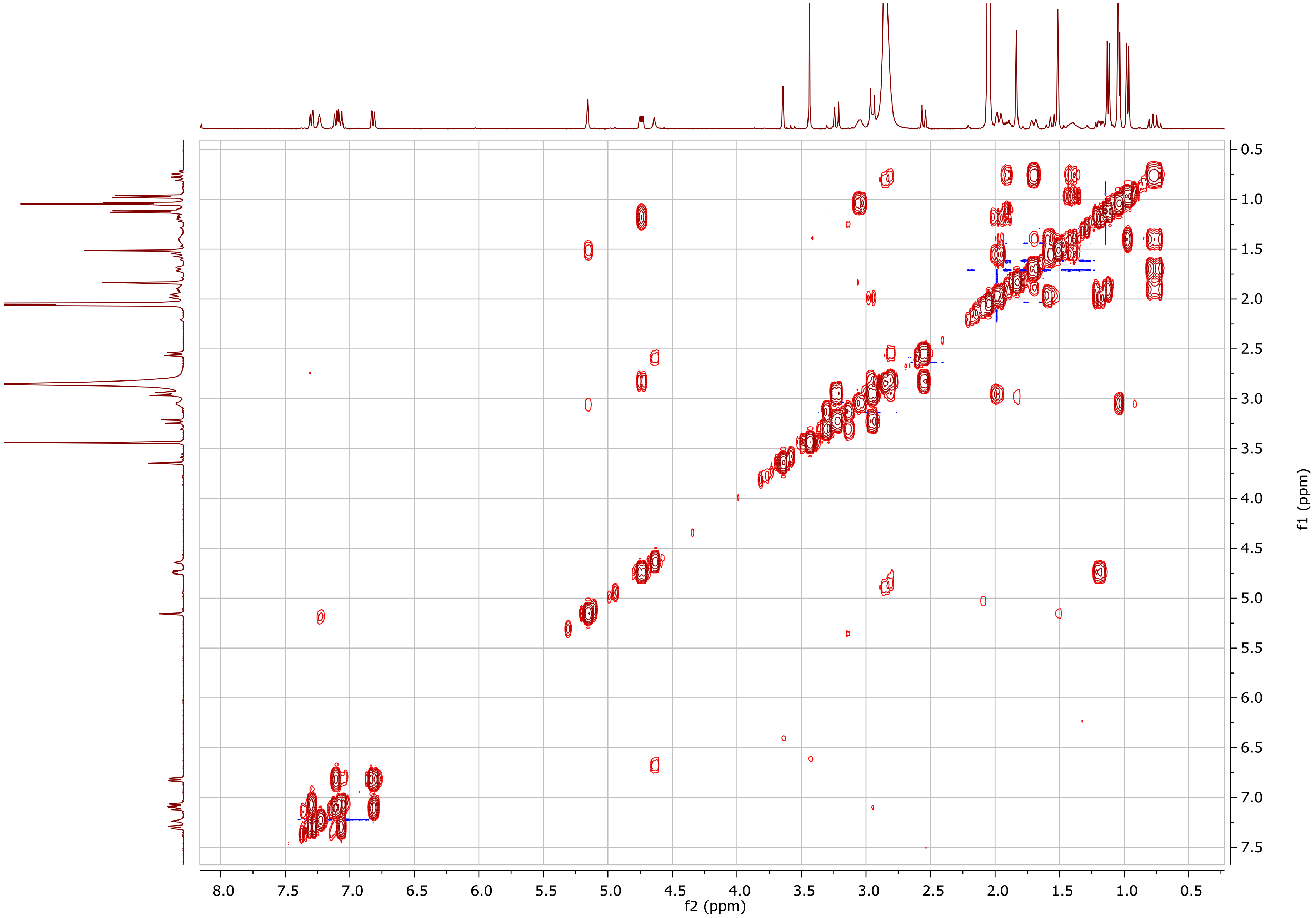

Figure S24. COSY NMR spectrum of **5** [500 MHz, acetone-d_6].

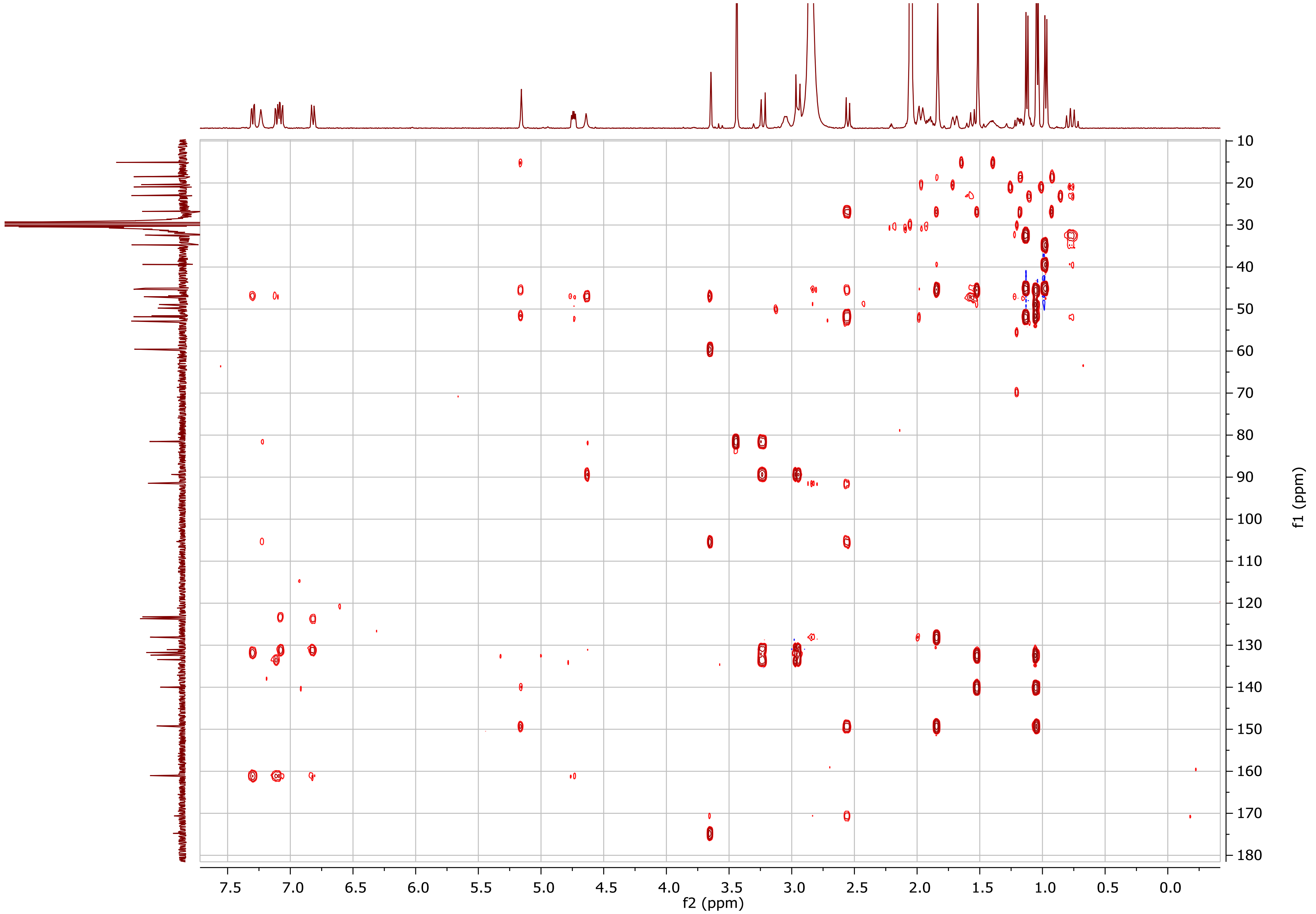

Figure S25. HMBC NMR spectrum of **5** [500 MHz, acetone-d_6].

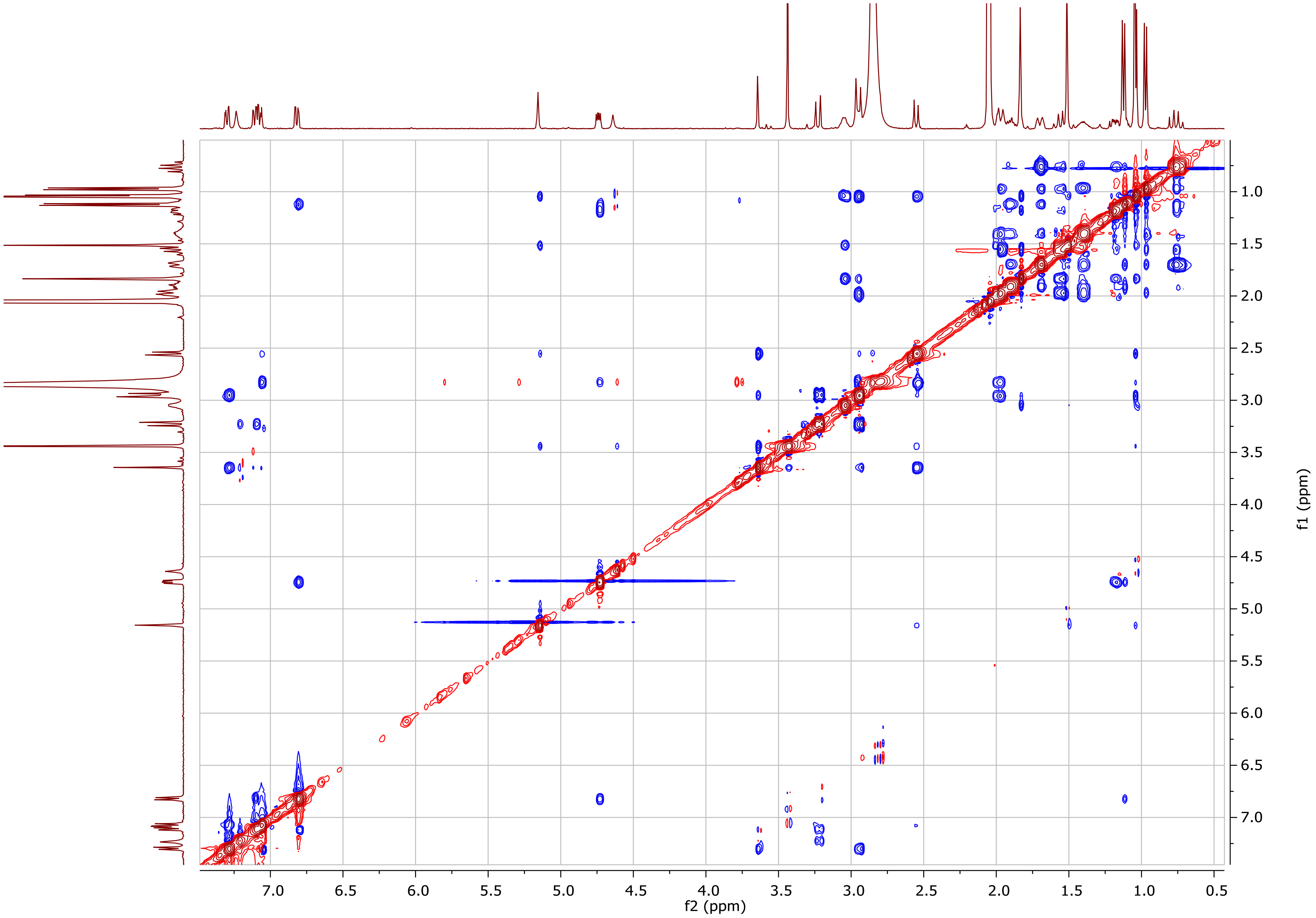

Figure S26. NOESY NMR spectrum of **5** [500 MHz, acetone-d_6].

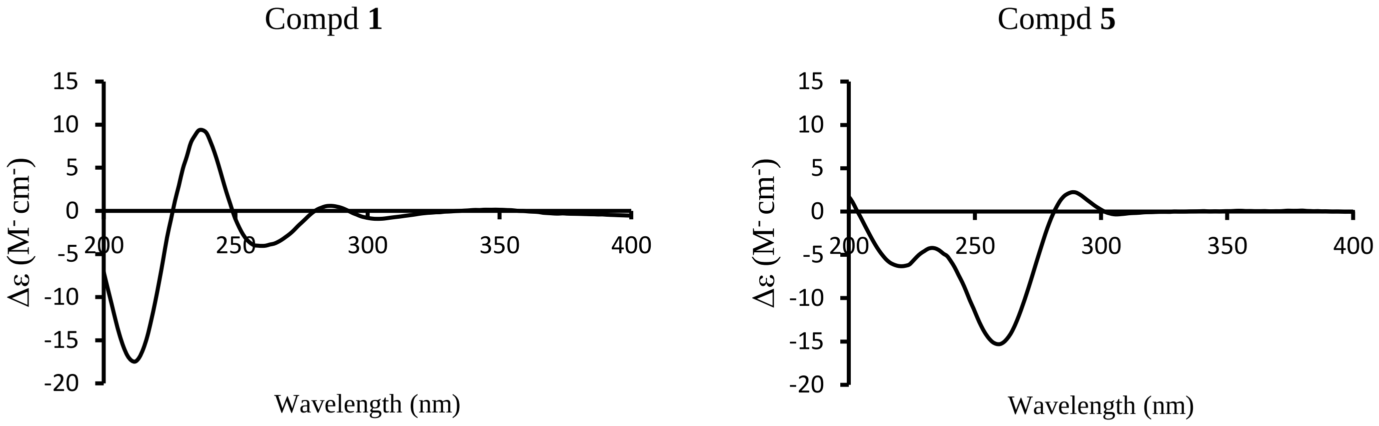

Figure S27. Experimental ECD spectra of compounds **1** and **5** in CH_3CN.

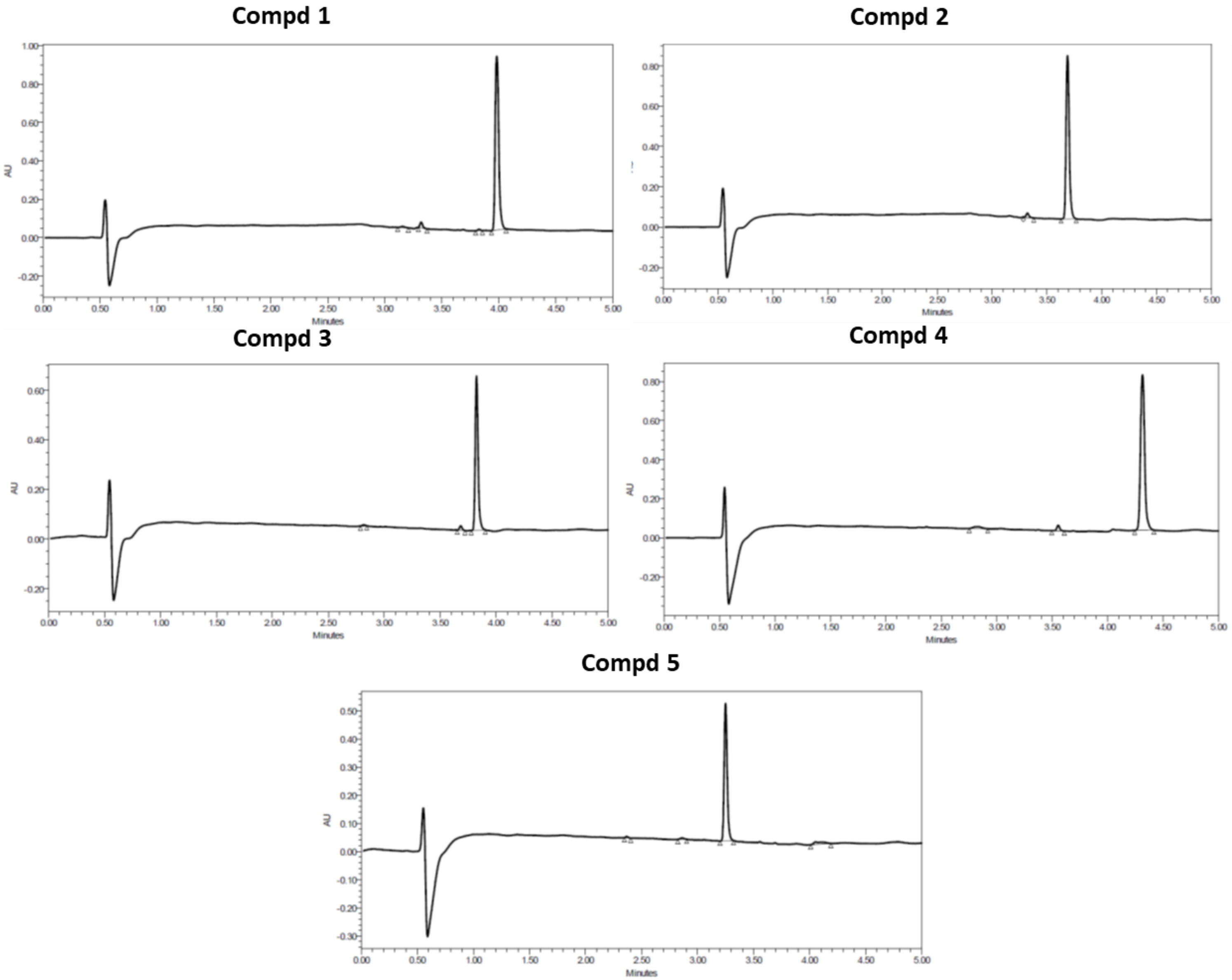

Figure S28. UPLC chromatograms of compounds **1–5** (λ 210 nm), demonstrating > 95% purity. Data were acquired via Acquity UPLC system using a BEH Shield RP18 column (Waters, 1.7 μm; 50 × 2.1 mm) and CH_3CN/H_2O gradient system that increases linearly from 15 to 100 % CH_3CN over 3 min.

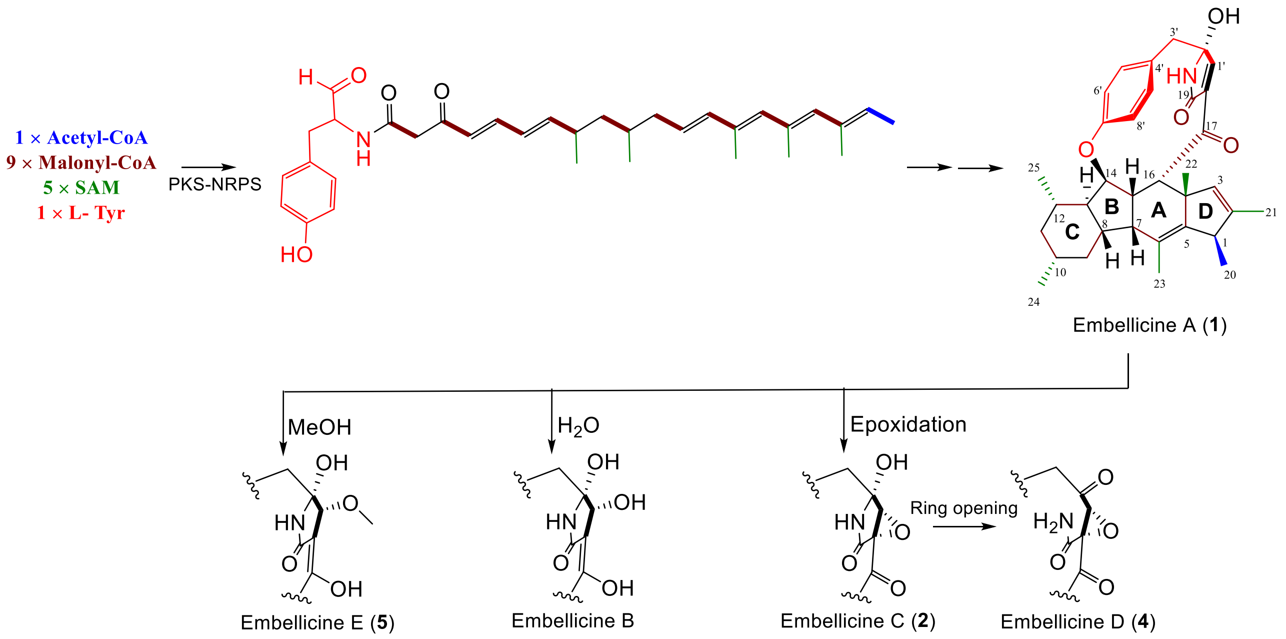

Figure S29. Proposed biosynthesis of embellicines with cyclopenta[b]fluorene (6/5/6/5) ring system.

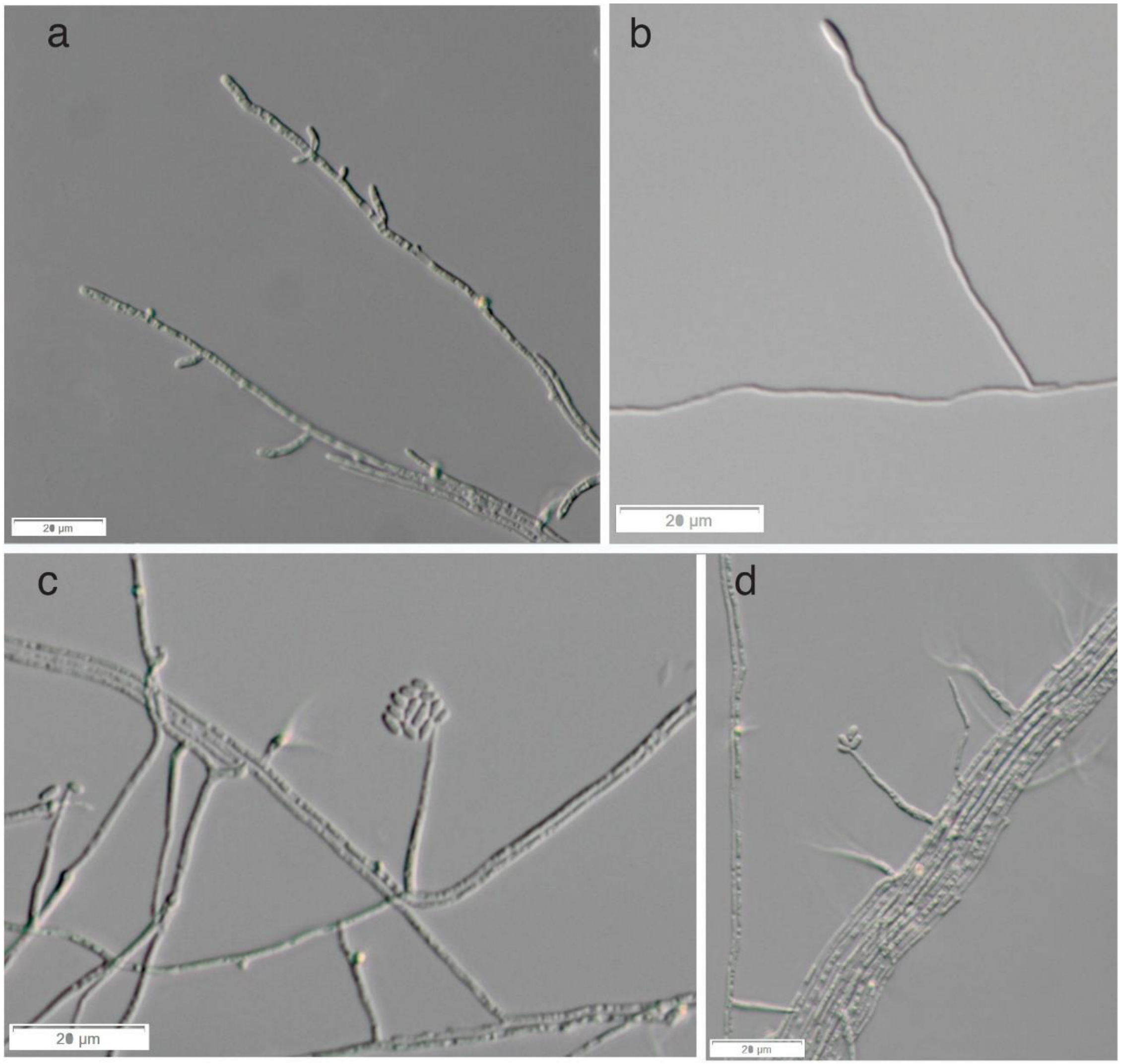

Figure S30. *Sarocladium* sp. (MSX6737). **a.** Vegetative hyphae, **b.** Cylindrical phialide arising from vegetative hypha, **c.** Conidia arranged in slimy heads, **d.** Conidia in slimy heads arising from ropes of vegetative hyhae. Measure bars = 20 μm.

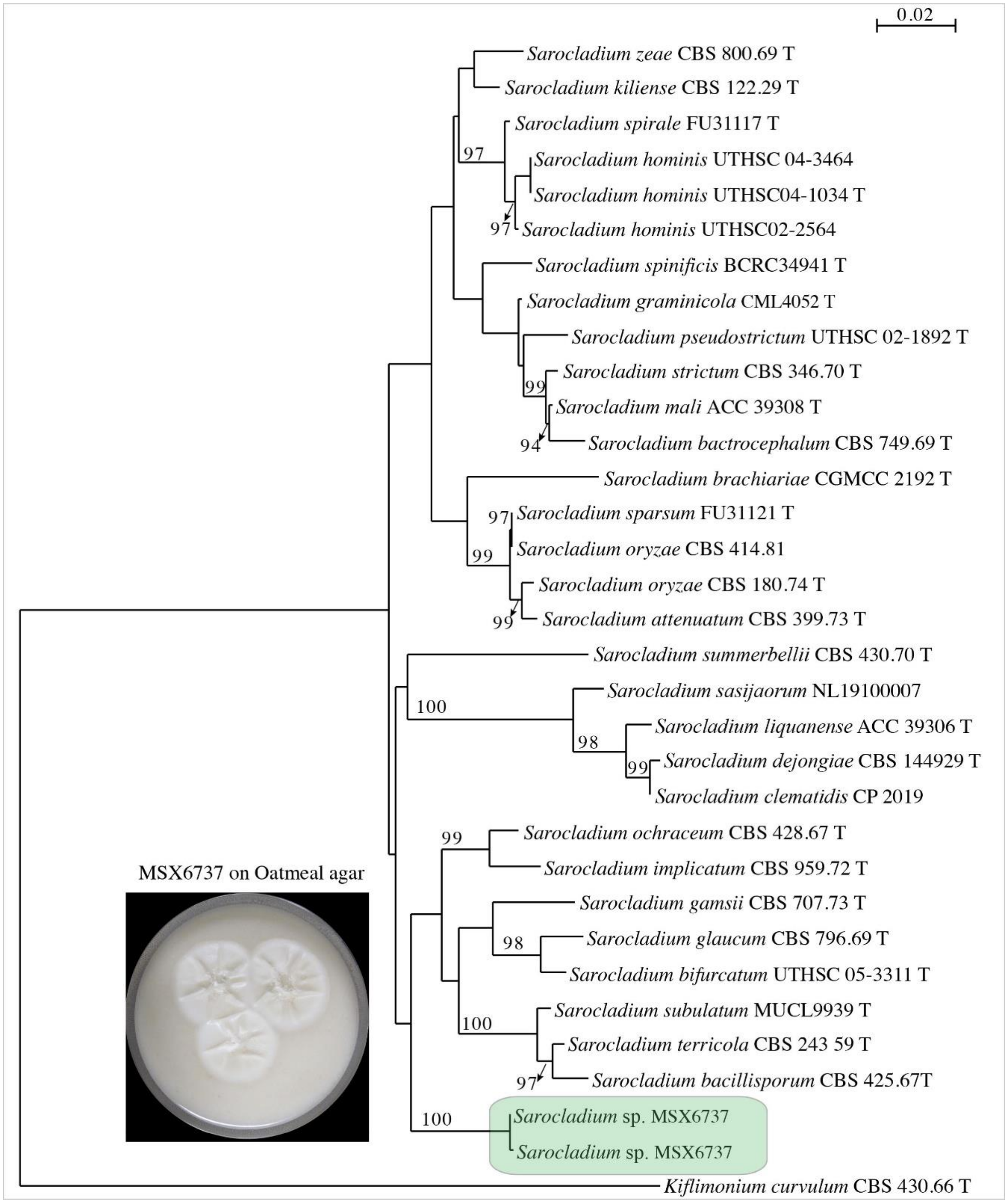

Figure S31. Molecular phylogenetics analysis of *Sarocladium* ITS-LSU sequences confirms MSX6737 can be referred to as *Sarocladium* sp., Hypocreales. Phylogram of the most likely tree (−lnL = 5042.959) from a ML analysis of 33 sequences based on the ITS and LSU region (1326 bp) using IQ-TREE. Numbers refer to UFBootstrap support values ≥ 90% based on 5000 replicates. Nodes ≥95 are considered strongly supported. A 10-d-old culture on oatmeal agar media is shown on the left. *Kiflimonium curvulum* CBS 430.66 was used as outgroup. Ex-type isolates are designated by a T. bar indicates nucleotide substitutions per site.

REFERENCES

(1) Wijeratne, E. M.; He, H.; Franzblau, S. G.; Hoffman, A. M.; Gunatilaka, A. A. *J. Nat. Prod.* **2013**, *76*, 1860-1865.
(2) Chen, Y.; Liu, Z.; Huang, Y.; Liu, L.; He, J.; Wang, L.; Yuan, J.; She, Z. *J. Nat. Prod.* **2019**, *82*, 1752-1758.
(3) Ebrahim, W.; Aly, A. H.; Wray, V.; Mándi, A.; Teiten, M.-H.; Gaascht, F.; Orlikova, B.; Kassack, M. U.; Lin, W.; Diederich, M.; Kurtán, T.; Debbab, A.; Proksch, P. *J. Med. Chem.* **2013**, *56*, 2991-2999.
